# Altruism or self-interest in tomorrow's veterinarians? A metric conjoint experiment and cluster analysis

**DOI:** 10.3389/fvets.2023.1044463

**Published:** 2023-04-05

**Authors:** Adele Feakes, Noel Lindsay, Edward Palmer, Paul Steffens

**Affiliations:** ^1^School of Animal and Veterinary Sciences, University of Adelaide, Roseworthy, SA, Australia; ^2^Entrepreneurship Commercialisation and Innovation Center, Adelaide Business School, Faculty of Arts, Business, Law and Economics, University of Adelaide, Adelaide, SA, Australia; ^3^Adelaide Business School, Faculty of Arts, Business, Law and Economics, University of Adelaide, Adelaide, SA, Australia; ^4^School of Education, Faculty of Arts, Business, Law and Economics, University of Adelaide, Adelaide, SA, Australia

**Keywords:** altruism, cluster analysis, empathy, metric conjoint analysis, other-orientation, prosocial motivation, self-interest, veterinary education

## Abstract

**Introduction:**

Altruism is considered a trait of veterinary and other health professionals, but the level of altruism in the veterinary profession is unknown. We designed a metric conjoint experiment to reveal other-orientation (an individual's caring concern for the wellbeing of others) and self-interest. We draw on the ‘Theory of Other-Orientation’, which states that individuals' decision-making heuristics can be impacted by their other-orientation independent of their self-interest. In patient-focused contexts, highly other-oriented or altruistic (veterinary) professionals may care too much for others and suffer immediate or cumulative financial and personal costs of such caring. At the same time, other-orientation can enhance job-related attitudes and outcomes, such as job satisfaction.

**Methods:**

In a metric conjoint experiment, Australian final-year veterinary, science, nursing, entrepreneurship, and engineering students rated eight job scenarios with orthogonally arranged high and low levels of three job characteristics (*n* = 586) to provide observed measures of other-orientation and self-interest.

**Results:**

A two-way MANOVA showed other-orientation or self-interest differed per discipline, but not gender. Veterinary (and engineering) respondents were less other-oriented than nursing respondents. Veterinary (and entrepreneurship) respondents were more self-interested than nursing respondents. K-Means cluster analysis confirmed four distinct profile groupings—altruistic/self-sacrificing, ‘both other-self’, self-interested and selfish—aligning with the discourse in the literature. Human nursing respondents stood out for the most members (50%) in the ‘both other-self’ profile compared to veterinary respondents (28%). Respondents of one of three veterinary schools stood out for the most members (19%) in the altruistic/self-sacrificing group.

**Discussion:**

Our metric conjoint experiment illustrates an alternative to ‘self-report’ items with Likert-scaled responses. Our finding of the ‘both other-self’ group adds to the literature, which considers that other-orientation and self-interest are separate constructs that are difficult to co-exist in individuals. This mix of traits is deemed helpful by organizational psychology scholars, for sustainability and wellbeing, especially for healthcare professionals involved in high-frequency and intense, patient-focused interactions. Our findings highlight the need for more research on the potential role of other-orientation and self-interest in veterinary school admissions processes, the hidden or taught curricula, job-related attitudes and beliefs, and wellbeing and professional sustainability in the veterinary sector.

## Introduction

Altruism features in veterinarians' views of professionalism (1) and is a motivator or characteristic of medical, nursing and other healthcare students (2, 3). Many in caring, especially patient-focused disciplines, view altruism as a professional behavior that is part of their “collective identity” (1, 4–10). Other-orientation is the extent to which individuals are concerned with the welfare of others (11) and becomes altruism when fulfilling an individual's other-orientation is unachievable without some cost to the self (12). Whereas, self-interest is the extent to which an individual is concerned with the welfare of their self. Other-orientation and self-interest are rooted in an individual's core values (13) and are considered enduring but separate traits.

The knowledge that value-based traits, such as other-orientation or self-interest, drive human decision-making permeates organizational psychology and behavior research (11, 12, 14–16). Personal values (2, 17) and traits, such as other-orientation, may predict an individual's decision-making regarding goals and intentional commitments, such as career choice, more than psychological attributes or demographics (2, 17–20). The extent of other-orientation and self-interest in the veterinary profession is unknown.

The continued value attributed by professionals as individuals or as a group, and by society, to high other-orientation could be considered contradictory and misguided in the context of paid healthcare careers (3, 21). We are concerned for veterinarians, as the medical social science literature reports that highly other-oriented medical doctors, without appropriate discernment and judgement, may over-respond in their day-to-day work, leading to a known path to apathy, burnout, and compassion fatigue (13, 21). Additionally, highly other-oriented persons may have insufficient self-esteem to withstand the fall in patient gratitude expression in the modern healthcare setting (3). Professionals in caring occupations such as veterinary medicine may care too much for their animal patients, owners, or team members and forget the immediate or cumulative financial or personal cost to themselves (leading to stress, burnout, and sometimes suicide) (13, 21–24). Informed by the literature, we take the position that a balance in other-orientation and self-interest in a patient-focused professional may be desirable for the sustainability of their wellbeing (16).

On the other hand, high other-orientation can positively impact individual-level outcomes and processes beyond helping in an organization (16, 25, 26); thus, the organizational behavior literature suggests that models of work behavior should consider an individual's levels of other-orientation and self-interest. For example, in performance appraisals or *ad hoc* feedback, other-oriented individuals more closely align in self-appraisal with their supervisors than more self-interested individuals (27), smoothing organizational processes and staff training conversations. That highly other-oriented individuals are less attracted to choices involving the prospect of personal gain and less discerning or disposed to engage with rational calculations involving costs, benefits, value, and risks, compared to individuals lower in other-orientation, also plays out in contexts not involving helping others (26) such as job attitudes and satisfaction. In three studies, job performance, prosocial behaviors, and personal initiative of highly other-oriented individuals were more a function of group-level attributes of the organization (e.g., justice climate). In contrast, those of highly self-interested employees were more a function of individual-level attributes (e.g., job attributes) (26). Similarly, in studies examining the relationship between beliefs about enriched job attributes^1^ and job satisfaction, such associations were weaker for highly other-oriented individuals (29). For the veterinary profession, with its current attraction and retention issues, the effect of other-orientation or self-interest on job attitudes and satisfaction is increasingly relevant as employers compete to attract and retain veterinary professionals.

Potential positive impacts of other-orientation on veterinary individuals and organizations motivate us to fill the gap in the literature on the levels of other-orientation or self-interest in entrants to or members of the veterinary sector. Thus, our primary aim in this study is to take the first step—a quantitative investigation of other-orientation and self-interest of veterinary respondents. We chose an experimental approach as decisions motivated by other-orientation or self-interest involve judgement when weighing up outcomes such as achieving benefit(s) to others or self. Strongly self-interested individuals are more likely to engage in “rational evaluation” of outcomes and valences for themselves. In contrast, strongly other-oriented individuals can only achieve purely altruistic goals by suspending rational self-interested processing to some degree (12). Though other-orientation and self-interest are defined in the literature as distinct constructs (12) rather than being mutually orthogonal, unipolar constructs (14), we take the position that while other-orientation and self-interest goals may be difficult to pursue simultaneously (12, 30), this may be possible and desirable (16). We chose to use a metric conjoint choice experiment that we designed to reveal respondents' relative levels of other-orientation and self-interest in rating a series of potential jobs differing on three attributes, available in their own occupation. Metric conjoint experiments predict eventual behavior more accurately (than self-rating items) (31). The participant's rating of each scenario harnesses decision-making and trade-off theory and draws on their multiple attitudinal and normative beliefs, engaging their automatic heuristic processes shaped by their own core beliefs (values) and valences (importance to them).

“Other-orientation” and “self-interest” are traits rooted in deep-held personal values, formed from human capital, gendered expectations, societal norms, and occupational socialization (32), all of which contribute to an individual's motivation to enter and persist in study and training for their chosen discipline. So it made sense to undertake our analysis using a sample population selected for (i) heterogeneity in personal values in relation to graduate career choices (33) and likely wide variance in other-orientation and self-interest, and (ii) shared characteristics with the veterinary profession, i.e., science and technology (engineering and science), delivery of services *via* private enterprise (entrepreneurship/business students) and delivery of patient centered medical care (nursing). We test our instrument on a pooled sample from the above five disciplines undertaking their study programs in a western educated, industrialized, rich and democratic (WEIRD) country (34). We present results for other-orientation and self-interest per disciplinary cohort, gender and for profile clusters that emerged from the data in subsequent cluster analysis. Our results particularly illustrate the diversity and four typical combinations of other-orientation and self-interest, across discipline and school-of-study based groups. Gender differences were not greatly apparent, especially in the veterinary respondents.

Our study will benefit researchers interested in measuring other-orientation and self-interest at the individual level in a career-relevant context. We propose a tool for (veterinary) scholars interested in incorporating a construct or measure of other-orientation or self-concern in (veterinary) social science quantitative research studies. Our study will inform scholars and practitioners by addressing other-orientation and self-interest (rather than gender) as factors in career choice. The cluster profile findings for our study population will benefit scholars and practitioners when considering the application of behavioral and organizational science theory—i.e., using multivariate rather than univariate-based approaches.

## Theoretical background and hypothesis development

Other-orientation and self-interest (35) reflect underlying values and deep or normative beliefs. Values, while often abstract, are known to be more stable (36), the basis of enduring individual traits, and less likely to change than the attitudes and behaviors they can directly or indirectly impact (36, 37). Values and deep or normative beliefs combine to inform an individual's decision-making heuristics and judgements of the utility (to them personally) of potential outcomes of their intentions^2^ or behaviors (31, 38, 39). Thus, variations in other-orientation and self-interest will be associated with core values, socialization or situational demands and constraints (35).

Rational self-interest is based on the welfare of the self as the primary motivator for the individual (40) and underpins Rational Economic Reasoning theory (26), amongst other theories. Rational self-interest is “thinking and acting in a manner expected to lead to an optimal or maximum result for a person based on a systematic consideration of the person's values and risk preferences” (27). Behavior is “…*self-interested if undertaken for the sole purpose of achieving a personal benefit or benefits…*” which may be tangible (e.g., money, a promotion) or intangible (e.g., community standing, group status). Self-interest in an individual stimulates information search and processing of self-relevant (self-rewarding) attributes, consequences and behaviors (35), with systematic (rational) weighing of personal costs and benefits when making choices and acting to pursue self-interested goals.

The Theory of Other-Orientation has materialized in the behavioral science literature in contrast and as a reaction to rational self-interest (rational economic reasoning)^3^. While other-orientation is defined as the extent to which individuals are concerned with the welfare of others (29), the core proposition of the Theory of Other-Orientation is two-fold: first, other-orientation involves the pursuit of others' welfare as the primary motivator for the individual; second, other-orientation is simultaneously less reliant on rational self-interested processing and subject to other drivers not common to self-interest (29) such as perspective taking ability (42), concern for others (43), identification with as interdependent and part of a social system (44), agreeableness (45), and high dispositional empathy (46). The Theory of Other-Orientation appears to be supported by neuroscience. Neuroscience-experimental studies using functional magnetic resonance imaging (fMRI) support that lower reliance on rational self-interested processing and greater dispositional empathy influence highly other-oriented decision-making and action. These fMRI-based studies reveal that other-oriented decisions (to the point of altruism) engage areas of the brain involved in empathic concern, pro-sociality, and self-interest but to different extents for different individuals (47–50). Further, an integration area in the brain was also identified^4^ where messages originating from brain areas responsible for empathy up-modulate other-oriented decision-making activity (48–50).

For decades, scholars and policymakers have been interested in other-orientation (OO) and self-interest (SI) in the individual in healthcare delivery occupations. More recently, other-orientation and self-interest constructs are emerging in organizational behavior and wellbeing literature. It makes sense that we draw attention to the roles of OO and SI in individual decision-making outcomes and organizational behavior modeling for the veterinary sector at the occupation level. We aim to answer the overarching research question driving this study: “*To what extent and in what combinations are other-orientation and self-interest evident in tomorrow's veterinary professionals?*”

### Other-orientation and discipline

Early studies reporting the level of other-orientation in caring occupations describe it as relatively homogeneous for individuals in education, occupational therapy, physiotherapy, speech pathology, and social work programs (51), as well as for faculty and staff of a large multi-campus health science center (52). Byrne (51) used Rushton's self-report altruism intention scale, and Valentine et al. (52) used modified recognized self-report altruism scales in this research (53, 54). Neither study included participants in “non-helping” occupations. This is in contrast to literature holding that heterogeneity amongst individuals in behavior encompassing both self-interested and other-regarding motivations will vary for the individual and the individual in different circumstances, e.g., in direct helping situations or the longer-term delivery or receipt of health care services (10, 55, 56). More recently, scholars have shown differences in “other-centeredness” and or “altruism” among prospective physicians (medical) and non-medical tertiary students (e.g., economics, arts, and natural science students) (9) and law/legal students (6, 9), but not business students (6).

Despite the minimal “field” evidence of other-orientated dispositions of health care professionals other than physicians (9) and nurses (2), we consider that those who have chosen veterinary career paths will be relatively high in other-orientation. We hold this as veterinarians decide to enter into their discipline underpinned by their intrinsic passion for animal care (57) and their inherent view of what veterinary professionalism, like health care professionals, includes being other-oriented, even to the extent of altruism (1). Though to the authors' knowledge, the level of other-orientation or self-interest in veterinarians or veterinary students is unreported in the literature.

We argue that levels of other-orientation and self-interest will differ for different discipline-based groups, influenced by members' core beliefs and situational, societal, group and gender identity factors. In the case of other-orientation, other enduring personal characteristics have been found to be associated with other-orientation: high perspective-taking ability (42), high concern for others (43), seeing themselves as interdependent and part of a social system (44), high agreeableness (45), and high dispositional empathy (46). While high agreeableness and dispositional empathy may attract (other-oriented) individuals to begin and continue training in patient-focussed disciplines, these traits would not necessarily confer an advantage in non-patient focussed disciplines (58). For these reasons, we propose the following hypotheses:

***Hypothesis 1a***. *The level of other-orientation for veterinary respondents will be more similar to respondents in another patient-focused discipline (nursing) and less similar to those in non-health-care disciplines*.
***Hypothesis 1b***. *The level of self-interest for veterinary respondents will be more similar to respondents in another patient-focused discipline (nursing) and less similar to those in non-health-care disciplines*.

### Other-orientation and gender

Overall, empirical studies hold that women are more other-oriented (often measured as altruism, with “costly helping scenarios”) (59). While early experimental studies (somewhat biased toward men) tested interactions with strangers showed men likelier to help others (32), later double-anonymous dictator experiments showed that donations triple when the recipient is a legitimate (i.e., a deserving recipient, not anonymous) charity (60). In a later modified double-anonymous dictator experiment also using strangers as recipients (without a known reputable charity involved), “demand curves for altruism” for men and women crossed. Men were more responsive to price changes (price sensitive)—i.e., more likely either to be perfectly selfish or perfectly selfless and helped more altruistically when they considered it “cheap” to do so. Women tend to be “equalitarians,” preferring to share evenly, helping more altruistically when considered costly, and being more price insensitive overall (61). In a subsequent double-round modified anonymous dictator game involving donation decisions to a known reputable charity, gender affected “altruistic giving” at the individual level and a social image signaling effect at the group (decision-making) level, with women donating more than men. In paired settings, mixed-sex groups gave the most. All-female and all-male pairs gave the least (62). Less altruistic partners (usually men) adjust their giving upward more than the more altruistic partners (usually women) reduce their giving (62). Gender^5^ effects on variables arise from identity role beliefs, human capital, and social norms (32, 64). The association of being female with greater altruism and altruistic behavior (61, 62, 65, 66) depends on the context, the personal cost, and other constructs that cannot be accounted for practically in every study (e.g., empathic concern, gender role identity beliefs,^6^ human capital, and societal norms).

Both the veterinary and nursing professions and student bodies are predominantly women. This might be related to women's motivation and their view of concern for others as a component of professionalism (1). Women are also known to be higher in agreeableness than men (67), and high agreeableness is characterized further by altruism, trust in others, cooperation, and empathy (58, 68). Additionally, women's other-orientation is more communally focused than men's (64).

As women are more communally focused, higher in empathy, and higher in agreeableness, we expect that women's level of other-orientation will be greater than men's, regardless of their disciplinary area. Other gender-related factors, such as gender role beliefs, social norms/societal gender role stereotyping, other motivations, or the structure of job opportunities for women (69), are also likely to contribute to other-orientation and self-interest, in addition to their agreeableness and empathy (67). Therefore, we hypothesize that:

***Hypothesis 2a:*** *The level of other-orientation for women will be greater than for men in our study population*.

As men are more agentically focused, lower in empathy, and lower in agreeableness, we expect that men's level of self-interest will be greater than women's. Therefore, we hypothesize that:

***Hypothesis 2b:*** *The level of self-interest for women will be less than for men in our study population*.

### Other-orientation, self-interest, veterinary discipline, and gender

If gender-related differences in other-orientation exist, this may confound the influence of other-orientation on caring occupation career choices. Women are more likely to choose nursing and other healthcare or helping professions than men (70–73). Women studying medicine, law, or business reported stronger attitudes consistent with altruism than men (6). In the healthcare sector, organization-based altruism was higher for women supervisors than men (52). Career choice for women is also partially attributed to motivational differences, societal gender role stereotyping, and the structure of job opportunities for women (69). Eagly (64) holds that gender effects on pro-social or other-behavior depend on the context of the behavior (64). They can be mediated by various contributors to gender role beliefs—hormonal processes, social expectations, or individual dispositions (values, traits). Men are more likely to be pro-social in an agentic context (behavior must be assertive, masterful, and dominant, e.g., involving danger). At the same time, women are more likely to be altruistic where the context is communally focused (friendly, unselfish, concerned for individuals/others, emotional, e.g. helping a distressed child) (64). Byrne (51) found, *via* interview and qualitative analysis, different altruistic reasons between men and women for entering helping professions (51). Men focused more on “society,” whereas women focused more on the “individual” person (51).

However, technological advances have lessened the importance of strength (to respond to danger) and have shifted requirements toward knowledge-based capability (increasing the potential for women to undertake agentically oriented prosocial behavior) in many occupations. Rapid medical and technical advances and increased pet ownership in developed countries have transformed veterinary clinical practice into one with considerable potential for women to undertake agentically oriented as well as communally orientated prosocial behavior. Unsurprisingly, the veterinary profession is now feminized as it attracts individuals with aligned agentic and communal values and identity beliefs.

We argue that with technological advances and increased pet ownership, the role of physical strength in agentic and communal identity beliefs has reduced as a drawcard for men into veterinary science as a career. We proffer that as agentic and veterinary communal identity beliefs are now more related to knowledge and technical skills, the playing field is more equalized such that men and women drawn to veterinary careers will be more similar in dispositional empathy. Therefore, we hypothesize that:

***Hypothesis 3a:*** *The level of other-orientation for women and men veterinary respondents will be similar*.

However, we argue that gender role beliefs and societal norms of the “man as the breadwinner” and self-interest in an individual will still be associated with the processing of self-relevant (self-rewarding) attributes, consequences, and behaviors (35). Further, as self-interest is a separate construct to other-orientation, we hypothesize that:

***Hypothesis 3b***. *The level of self-interest for men veterinary respondents will be greater than for women veterinary science respondents*.

### Profiles of other-orientation and self-interest

Although decision rule patterns are likely to vary among respondents, they are expected to be similar enough such that cluster analysis techniques will reveal groups of respondents with similar relative importance beliefs (i.e., similar decision-making models). For example, we would expect individuals high in other-orientation to place greater importance on the opportunity to help others as a job characteristic (in their chosen discipline) compared to the importance to them of monetary remuneration or lower work effort. We would expect a more self-interested person to prefer lower work effort and/or higher financial compensation for their work since self-interest in an individual is known to stimulate judgement of self-relevant (self-rewarding) benefits, consequences, or outcomes (12, 35).

However, we proffer that individuals in a population will fall into profiles that differ in the balance of their priority for other-orientation and self-interest. As per Le Grand, prospective professionals, whatever their discipline, will not be pure “knights” (with other-orientation dominant over self-interest) or “knaves” (purely self-interested), but different mixes of these characteristics. We contend that some individuals will be more “knight” than “knave” and vice versa (10) or a combination thereof, as individuals are inherently not equally motivated to pursue their self-interest. We also contend that people are not so different that discrete and interpretable clusters of respondents will exist in a study population (29). Further, we take the position that while other-orientation and self-interest motivations and goals may be difficult to pursue simultaneously (12, 30), it is possible and desirable (16). Therefore, we hypothesize that:

***Hypothesis 4:*** *Our study population will have distinct interpretable profiles of different combinations of other-orientation and self-interest, including a profile of individuals who are both other-oriented and self-interested*.

### Cluster profiles and discipline

Diversity in other-orientation and self-concern across individuals choosing veterinary science is unclear. The literature tells us that nurses and veterinarians, while highly motivated to help others when selecting and training for their career paths, have heterogeneous motivations (2, 3, 57). That is, different motivations drive people toward the same career choices (63, 74). Further, given the diversity in views of professionalism (1) and identity role beliefs and motivations (57), we expect diversity in other-orientation and self-concern in those who choose a career in veterinary science. Therefore, we hypothesize that:

***Hypothesis 5:*** *There will be differences in the proportional representation of identified cluster-based profiles of veterinary respondents to respondents of other disciplines*.

### Cluster profiles and veterinary program

Given the heterogeneity in motivations of veterinarians when selecting and training for their career paths we would expect that different veterinary schools (i.e., in different universities) may attract applicants who differ in their core values, identity beliefs, societal norms and other factors that are reflected in differences in their other-orientation and self-interest (1, 57). Further, the extended time in a study program such as veterinary science, and veterinary study programs being very linear and heavy in core course requirements, especially exposes individuals to the local community norms, and hidden and taught curriculum. We would expect differences between universities and veterinary schools to be reflected in the graduands' core values and identity based beliefs, and potentially then in differences in their other-orientation and self-interest, during and by the end of their study program (75). Therefore, we hypothesize that:

***Hypothesis 6:*** *There will be differences in the proportional representation of identified cluster-based profiles of veterinary respondents from different institutions*.

## Measurement of other-orientation and self-interest

Three broad approaches characterize the measurement of other-orientation and its “companion” construct, self-interest. First, self-report surveys have had considerable use (6, 51, 76, 77) but are not recommended (46). Psychometric scales exist for altruism (78), and instruments that measure self-concern or self-interest *via* the importance of extrinsic rewards to the respondent exist. However, measures using self-report Likert-like response options are susceptible to acquiescence bias (79), positive-response, and common method bias arise when determining the level of other-orientation or self-interest (11, 80). Further, and despite the advice of Ajzen and Fishbein (31), self-report scale-based measures of other-orientation and altruism are often mislabeled when they would be more aptly named pro-sociality scales (6, 51, 76, 77). A second approach uses open-ended text survey questions analyzed for content (81) or *via* interview approaches, particularly in the context of career choices, where researchers ask stated reasons for chosen careers, with subsequent coding of text entries or interview transcripts (82). A third approach is the choice experiment: in which the participants rate a number of scenarios dichotomously or on a metric scale. The rating involves trade-off decision-making, drawing on the participant's multiple attitudinal and normative beliefs. These predict eventual behavior more accurately (than self-rating items) (31). One example involves “dictator games” (60, 61, 83). Another example from the health economics policy literature involves laboratory-based experiments to determine other orientated and self-interested behavior (using prospective physician and non-physician student participants) (9, 84). Entrepreneurship scholars interested in other-regarding or pro-social values of potential entrepreneurs have used the metric conjoint (within-subject) experiment (mCE) (85, 86). The mCE identifies attributes' relative contribution(s) (utility) to respondents' preference ratings for objects or hypothetical scenarios (87). Differences in the utility of job attributes reflect an individual's beliefs and values as this influences their decision-making rules. For example, some individuals will value the opportunity to be other-oriented (even to the point of altruism) in a series of hypothetical scenarios. This will be of prime importance to them compared to other characteristics of the hypothetical scenarios. The mCE tool models real-time decisions (rather than relying on self-reports) and predicts actual behavior well (88) when employed correctly and using realistic decision profiles. The mCE minimizes acquiescence bias by respondents undertaking decision-making behavior in scenarios requiring trade-off decisions—underpinned by decision rule and microeconomic utility theories (88–93). Other advantages of the mCE are its deliverability to large numbers of participants and its ability to generate continuous variables for statistical analyses.

We utilized this third measurement approach by conducting a metric conjoint (with-in subject) experiment [see, for example, (86, 89)] to reflect participants' other-oriented and self-interested beliefs and values. We used *K*-means clustering to reveal interpretable profiles that align with the background literature discussed above. We then compare and discuss these results against the literature. Last, we summarize our contributions and note implications for policy, education, and further research.

## Methods

### Sample and instrument

We purposefully sought participants in “helping” and “non-helping” vocations (94, 95) using stratified and convenience (non-probability) sampling of engineering, entrepreneurship, nursing, science, and veterinary graduands at one institution, and veterinary graduands at two other institutions. We chose five of Australia's seven veterinary schools for our initial sample frame. We chose these five to represent the sample frame and optimize imposition on the student population. Two of these schools, however, were changing their programs (from Bachelor's to Master's level), leaving three participating veterinary schools in the sample frame. These three schools had different admissions processes, were geographically distant, and were spread across Australia—from East to West.

Participation in the paper-based survey was primarily during allocated class time in final or penultimate semesters by arrangement with the program or course-co-ordinators of the participating study programs. The survey was delivered and introduced by staff who did not teach the participants—in the case of non-veterinary participants, by the principal investigator (the primary author); and in the case of veterinary students, by non-teaching staff. Participants received a verbal and written preamble introducing the study's importance and purpose, assuring their participation was voluntary and anonymous and that data management was confidential. Respondents undertook a metric conjoint experiment and then answered questions to collect information on their demographics and self-report items. As all variables were from a single source, we chose to mitigate the potential for common (correlated) method bias in the data by implementing recommendations in the research design and analysis phases. In this regard, we sourced the metric conjoint experiment and the Likert scale self-report items from the literature, positioned the mCE experiment first, and placed items requiring lower cognitive load at the end of the survey (80, 96).

We obtained 639 responses (Supplementary Table 1), comprising 614 on paper (2015, 2016, and 2017) and 15 online (2017) (Qualtrics^®^) and representing ~52% of enrolled students in the classes surveyed. Response bias was deemed minimal for entrepreneurship and nursing cohorts, with 70 and 79% response rates, respectively. Response rates from engineering and science students were acceptable. The actual class attendance rate for engineering and science students was close to the participation rate due to students being on placements or classes not requiring physical attendance. We retained in our study population both domestic and international students despite the finding by Xu et al. (63) of greater altruism scores for medical applicants^7^ who spoke a language other than English at home (mainly Asian or Southeast Asian backgrounds) (63). We eliminated cases in three stages. First, we removed 20 cases with >5% missing variables, a response invariance across variables (indicating speeders), or missing responses for gender (three cases). Second, cases were removed for whom values in the reliability check (repeated scenario 4) for the conjoint profiles were ≧3 (93). Third, from the retained cases (*n* = 594, Supplementary Table 2), we removed eight cases with outliers of the dependent variables required to satisfy the necessary assumption testing for the MANOVA procedure. The final sample for our study population comprised 586 respondents (median age 23 years, 67.7% women, 19.6% international student enrolment status; Table 1).

**Table 1:** Demographic profile of respondents after data hygiene and removal of eight outliers for the dependent variables of the MANOVA (*n* = 586).

	**Total**	**Median age**	**International % (*n*)**	**Male % (*n*)**	**Female % (*n*)**
Engineering 1	59	22.0	35.6% (*n* = 21)	83.1% (*n* = 49)	16.97% (*n* = 10)
Entrepreneurship 1	66	24.0	77.3% (*n* = 51)	51.5% (*n* = 34)	48.5% (*n* = 32)
Nursing 1	171	21.0	6.9% (*n* = 12)	9.2% (*n* = 16)	90.8% (*n* = 157)
Science 1	56	22.0	21.4% (*n* = 12)	58.9% (*n* = 33)	41.1% (*n* = 23)
Veterinary science (ALL)	232	24.0	8.2% (*n* = 19)	24.6% (*n* = 57)	75.4% (*n* = 175)
**Total**	**586**	**23.0**^**∧**^**(*****n*** **=** **583)**	**19.6% (*****n*** **=** **115)**	**32.3% (*****n*** **=** **189)**	**67.7% (*****n*** **=** **397)**
Veterinary science 1	84	24.0	8.3% (*n* = 7)	25.0% (*n* = 21)	75.0% (*n* = 63)
Veterinary science 2	98	23.0	0.0%	25.5% (*n* = 25)	74.5% (*n* = 73)
Veterinary science 3	50	24.0	24.0% (*n* = 12)	22.0% (*n* = 11)	78.0% (*n* = 39)

1 University A, 2 University B, 3 University C.

^∧^Skewness = 3.318, kurtosis = 14.390, minimum age 19 years, maximum age 58 years.

The generally higher median ages of Veterinary Science and Entrepreneurship respondents reflected that these were from extended bachelor and postgraduate veterinary programs or combined undergraduate and postgraduate courses (in the case of Entrepreneurship). Of the international students, 51% were women compared with 71% women domestic students. Nursing had the highest representation of women, and engineering had the most men.

### Measures

#### Other-orientation and self-interest

We designed our metric conjoint experiment (mCE) to “reveal” respondents' other-oriented and self-interested beliefs and values in an experimental context—as their beliefs and values contribute to their decision-making in the experiment. We created a context relevant to our multi-disciplinary sample population (i.e., employment or job search scenarios). We would expect highly other-oriented individuals to place importance on the opportunity to help others as a job characteristic. We expected highly self-interested individuals to prioritize financial remuneration (income, salary) and lower work effort or intensity. Work effort is defined as the product of time spent working and an index of work intensity (85). We included work effort in our metric conjoint experiment, considering that it is generally accepted that people are averse to work (97). This is congruent with rational self-interested reasoning and will be reflected in the negative utility of work effort for them. We expect “revealed other-orientation” and “revealed self-interest” measures produced from the mCE to correlate positively with related self-report measures (which we included in our survey instrument as nomological validity checks).

Our mCE involved participants *rating* the attractiveness of eight hypothetical professional positions (jobs)—conjoint profiles—that simulate real-life job characteristics. The rating task forced them to trade off the importance to them in a job of (a) the opportunity to undertake other-oriented (including altruistic) behavior, (b) income earning potential, and (c) work effort when rating each job profile. In this way, the experiment forced respondents to trade off key employment characteristics. Each profile had a different combination of high and low levels of the abovementioned attributes (Table 2). We used high and low levels as plausible variations for the decision environment while maintaining face validity (98). Using SPSS^®^, we generated the eight-profile fully “orthogonal array” [(two levels)^3^ = 8] with altruism, income, and work attributes. We then randomized the profile order to minimize order effects.

**Table 2:** Conjoint measures: attributes, definitions, and levels.

**Attribute**	**Definition**	**Levels**	
Other-orientation (altruistic)	When we act to promote someone else's welfare, being selfless, unselfish, or “other-directed,” even at a risk or cost to ourselves.	+1	HIGH degree that the position would allow you to help others who are less fortunate than you are, typically *via* the provision of free, at-cost, or cheap services, or *via* your personal effort, risk, or time
		−1	LOW degree that the position would allow you to help others who are less fortunate than you are, typically *via* the provision of free, at-cost, or cheap services, or *via* your personal effort, risk, or time
Income	The monetary rewards that you want for your work (so you can buy the things you like)	+1	HIGH level of monetary rewards compared to the average for people of your age, education, and experience.
		−1	LOW level of monetary rewards compared to the average for people of your age, education, and experience.
Work effort	The relative amount of work (time or intensity) required and its related impact on your time for other non-work activities	+1	HIGH relative amount of work (time or intensity) required and impacts on your time for other non-work activities
		−1	LOW relative amount of work (time or intensity) required and impacts on your time for other non-work activities

At the beginning of the study, the mCE instrument included the eight-profile orthogonal array used twice (repeated with the attributes in mirrored order, i.e., work, income, then altruism) to allow a test-retest measure of the reliability of each profile (99). Analysis of these responses revealed high correlations and non-significant paired sample *t*-tests between the mirrored items. Feedback from the 2015 participants was that the mirrored profiles annoyed them because of the obvious repeating questions. To reduce this imposition on participants in subsequent data collections, we included only a mirror of profile four as a test-retest measure of reliability.

**Figure 1:**
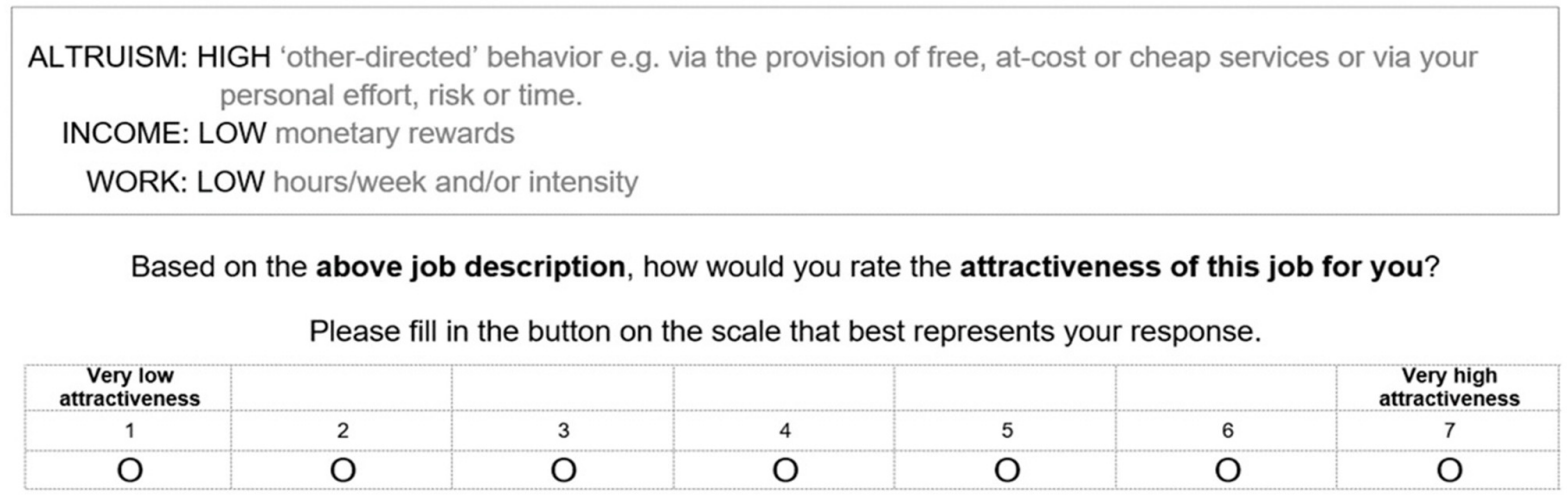
Example profile showing attribute prompts and the seven-point rating scale for one of the hypothetical job profiles in the conjoint experiment.

In all deliveries of the survey, two versions (one a reverse order of the other) of the mCE instrument were randomly assigned to each group of participants to mitigate against scenario order effects (99). We provided clear definitions of assumptions and the attributes in the experiment preamble to control for differing perceptions of personal wealth and occupational cohorts. We asked participants to assume that for each position profile: (i) Your special skills/expertise acquired in their current degree are required; (ii) The availability of the position is within 2 years of graduation^8^ and is in today's economic environment; (iii) You have become free from debt related to university/studying (e.g., from the Australian Government funded Higher Education Contribution Scheme (HECS) which is a low-interest loan to support Australian UG students pay for their university tuition fees); and (iv) All other aspects of the positions are the same for you. We provided an example response (Figure 1) to familiarize respondents with the task (98). We provided attribute prompts within each job profile, and we asked respondents to rate the attractiveness for them of each of the eight job profiles on a seven-point scale, anchored by “very low attractiveness” (“1”) to very high attractiveness (“7”).

Analysis of the interval-scaled preference rating data derived from the mCE uses an “*a posteriori*” decomposition of the decision process of each respondent (100). The “decomposition” of respondents' rating data reveals the beliefs and personally held decision policies or “rules” used by each respondent in arriving at their rating judgements. The technique provides full multilinear (main effects plus interactions) decision models based on multiple regression error theory (87). Metric conjoint analysis (“mCA”) is able to be conceptualized as a non-parametric statistical procedure that is a counterpart to traditional multiple regression analysis but with two distinguishing outputs: the (i) *utility* of each attribute level; and the (ii) *relative importance* of each attribute in the respondent's decision process. Utilities (or “part-worth utilities”) are typically derived using Ordinary Least Squares (OLS) dummy variable regression analysis (101). The respective β*s* in the equation below are the regression coefficients representing the respondent's interval-scaled *part-worth utility* for each attribute. The equation describes the full model for this research for the upper level of each attribute (value = 1.0). J represents the respondent judgement rating, μ represents a constant, and X_1_, X_2_, and X_3_ represent the three attributes—other-orientation (OO), income (INC), and work effort (WK).


$$\begin{array}{l} J=\beta_{1}X_{1}+\beta_{2}X_{2}+\beta_{3}X_{3}+\;\mu \end{array}$$

The relative importance of each attribute for the participant indicates the degree of “trade-off” an individual is willing to consider and will smooth out effects related to optimism/pessimism or acquiescence biases. Increasing the relative importance of one attribute will come at the cost of the relative importance of the other attributes. To calculate the “*relative importance*” of each attribute, we (1) determined the part-worth range for each attribute; (2) summed the absolute values of these ranges, and then (3) calculated for each attribute, the percentage of its part-worth range over the sum of all attributes' part-worth ranges as illustrated by the following equations.


$${\text{ATTRIBUTE}}_{\text{RI}}=\frac{\text{range of ATTRIBUTE}\;\text{part-worths}}{\sum \text{|ranges| ATTRIBUTES}}$$

As such, we used the following equations to calculate the relative importance score of each attribute:


$$\begin{array}{l} {\text{ OO}}_{\text{RI}}=({\text{OO}}_{\text{pw2}}{\text{-OO}}_{\text{pw1}})/[\text{abs}({\text{OO}}_{\text{pw2}}{\text{-OO}}_{\text{pw1}})+\text{abs}({\text{INC}}_{\text{pw2}}{\text{-INC}}_{\text{pw1}})+\text{abs}\;({\text{WK}}_{\text{pw2}}{\text{-WK}}_{\text{pw1}})]\\ {\text{INC}}_{\text{RI}}=({\text{INC}}_{\text{pw2}}{\text{-INC}}_{\text{pw1}})/[\text{abs}({\text{OO}}_{\text{pw2}}{\text{-OO}}_{\text{pw1}})+\text{abs}({\text{INC}}_{\text{pw2}}{\text{-INC}}_{\text{pw1}})+\text{abs}\;({\text{WK}}_{\text{pw2}}{\text{-WK}}_{\text{pw1}})]\\ {\text{ WK}}_{\text{RI}}=({\text{WK}}_{\text{pw2}}{\text{-WK}}_{\text{pw1}})/[\text{abs}({\text{OO}}_{\text{pw2}}{\text{-OO}}_{\text{pw1}})+\text{abs}({\text{INC}}_{\text{pw2}}{\text{-INC}}_{\text{pw1}})+\text{abs}\;({\text{WK}}_{\text{pw2}}{\text{-WK}}_{\text{pw1}})] \end{array}$$

For use in the multivariate means comparisons, regression modeling and the *K*-means cluster analysis, we calculate the composite for self-interest:


$$\begin{array}{l} {\text{SI}}_{\text{RI}}={\text{INC}}_{\text{RI}}-{\text{WK}}_{\text{RI}}9 \end{array}$$

^9^

When we report results for “other-orientation,” we use the above score calculation for OO_RI._ When we report results for “self-interest,” we use the above score calculation for SI_RI._

#### Gender, nationality, and age

Using checklist choices, we obtained categorical data from each participant on gender (where 1 equals female and 0 equals male) and nationality (where 1 = international enrolment and 0 = domestic student enrolment). We obtained age as free text entry scale data. We included these variables as we were interested in gender as a key explanatory variable, and we wished to be able to account for age and international student enrolment status (noting that human capital and social norms are likely to differ for domestic students).

#### Discipline

Using checklist choices, we obtained categorical data on participants' discipline of study and university. We then transformed the multi-categorical discipline variable into five dichotomized variables representing engineering, entrepreneurship, nursing, science and veterinary disciplines. We transformed the multi-categorical variable of university into three dichotomized variables representing the three participating universities, respectively. We used the university categorical variables to account for the effect of veterinary school-of-study on the dependent variables for veterinary respondents from university 2 and university 3.

#### Prosociality and importance of career-derived income (self-reported)

We included in our survey instrument 10 items^10^ used in similar study populations: eight items capturing “altruistic” attitudes and beliefs (Cronbach α = 0.862) and two items capturing the importance of income to the respondent in their career and in general (6, 63). The eight items represented subdimensions of professionally focused pro-sociality, three of which captured self-rating of the propensity to enact behaviors selflessly (for no reward) to promote or benefit another's welfare (4, 102, 103). For the eight items for our study population, internal reliability was acceptable [α 0.742 (95% CI: 0.709, 0.773); Omega 0.726 (95% CI: 0.709, 0.773)]. Based on confirmatory factor analysis results, we used seven items to construct a composite self-reported “prosociality” variable. We retained both income importance items to create a composite variable called “income importance.”

#### Cluster analysis

Cluster analysis is a multivariate interdependence technique that classifies cases into a small number of mutually exclusive groups based on similar configural profiles of certain variables, such as personal and environmental attributes (104). We used *K*-means clustering to determine if discrete and interpretable clusters of respondents exist in our study population. *K*-means clustering uses “centroids” for “*K*” different randomly-initiated points in the data and assigns every data point to the nearest centroid. After assigning every point, the centroid is moved to the average of all the points assigned. Cluster analysis appears to be an appropriate strategy for analyzing other-orientation and self-interest in individuals because of past theory (11, 15, 26, 35, 105). In our study, we wish to “type” or profile our respondents per their revealed other-orientation or self-interest relative importance scores from our metric conjoint experiment. Decision rule patterns will vary among individuals but will be similar enough that cluster analysis of the responses should reveal respondent groups with similar decision-making models and beliefs. Further, after assigning individuals to profiles, proportional analysis of such profile groupings across occupational cohorts and gender identity groupings can be undertaken.

### Data analysis procedure

To prepare for the analysis, we Winsorized the age data for our study population, bringing upper outliers into the 32-year-old category to achieve a closer to normal distribution as assessed by skewness and kurtosis (104, 106). There were no missing values for the key variables of interest (*n* = 586), i.e., the part-worth utilities from the metric conjoint experiment, gender, discipline, and international student status. There were no cases with 5% or more missing values for all measures used in the analysis (Littles test: Chi-Square = 66.436, df = 84, *p* = 0.921). Before composite formation, we replaced the small amount (1–2 cases) of missing data for several of the self-report continuous items (6) by applying the EM procedure in SPSS^®^. We used the Spearman Rho correlation as many of our variables were ordinal. We used metric conjoint experiment data analysis (SPSS v23.0^®^) to compute the utility (part-worth) scores for each level of the three attributes. We used the compute variable function in SPSS^®^ to compute the relative importance scores for each variable (other-orientation, income importance, and work effort relative importance). We used descriptive statistics and Gabriel or Games-Howell *post-hoc* comparison tests for ANOVA and MANOVA procedures undertaken for this study.

Given the conceptual and methodological relationship between our dependent variables, Other-Orientation (OO_RI_) and Self-Interest (SI_RI_), we ran a two-way between-groups multivariate analysis of variance (MANOVA) to investigate gender and disciplinary group differences in the two dependent variables simultaneously. The MANOVA enables better control or adjustment for the increased risk of a Type I error (107). We undertook assumption testing to check for normality, linearity, univariate and multivariate outliers, homogeneity of variance-covariance matrices, and multicollinearity, with no serious violations noted before and in the MANOVA modeling process (104, 107). First, we used Mahalanobis distance and scatter plots to identify and remove eight outliers in the relationship between OO_RI_ and SI_RI_. The skewness of OO_RI_ (−0.173) and SI_RI_ (−1.862) and kurtosis of OO_RI_ (0.587) and SI_RI_ (4.445) improved after removing the eight outliers (skewness −0.129, −1.510; kurtosis 0.574, 2.883, respectively). The correlation between OO_RI_ and SI_RI_ of −0.377 [−0.444, −0.306], *p* < 0.001 increased in magnitude after removal of the eight outliers (*N* = 586) to *r* = −0.524 [−0.580, −0.462], *p* < 0.001. Levene's test for homogeneity was satisfied by both OO_RI_ (*p* = 0.112) and SI_RI_ (*p* = 0.565). For the final multivariate model (*N* = 586), homogeneity of the covariance matrix (Box test) was not found (*p* < 0.001),^11^ but this is not unexpected with large sample sizes (107). We proceeded with the MANOVA as all other assumptions we tested were acceptably achieved, and our study population were well over >200 (104, 107). To support our findings in the MANOVA, we ran a *post-hoc* multivariate hierarchical linear regression (MHLR) model with the stepwise introduction of additional independent variables and interaction terms, with other-orientation and self-interest as the dependent variables. We undertook this MHLR modeling using MPlus v8.7^®^ using robust maximum likelihood (MLR) estimation, which takes into account the possibility of non-normality and heteroscedasticity in the data. This is achieved by using a robust estimator of the covariance matrix, which is less sensitive to the presence of outliers and non-normality in the data than the traditional maximum likelihood estimator. We included additional demographic variables to gender and discipline, which we considered may act as proxies for human or social capital or role identity beliefs and explain additional variance in the dependent variables to that explained by discipline or gender. We then added two-way interaction terms that were thought likely to be relevant to the model, then removed in stepwise re-runs of the model, based on the highest *p*-value, one interaction term for each dependent variable, until only significant interaction terms remained. We also included two attitudinal composite variables formed from Likert-like response items—Prosociality and Income Importance. We report the Standardized (STDYX) Model Results for estimates, dependent variable correlations, and variance explained (*R*-square) by the independent variables in the dependent variables.

Finally, we undertook a *K*-means clustering analysis of the dependent variables formed from relative importance scores for each variable (other orientation, income importance, and work effort importance; SPSS v28.0^®^). For the *K*-means clustering, we set iterations to a maximum of 20 and convergence at 0.0. We repeated the clustering four times with the dataset reordered (i.e., ascending, and descending case number; ascending and descending gender). We used *post-hoc* one-way ANOVA to verify that the cluster profiles were significantly distinguishable. We conducted *post-hoc* frequency analysis per vocational cohort and gender identity groupings.

## Results

### Preliminary descriptive results for variables used in the study

Mean scores (with standard deviations where appropriate) and correlations for the dependent and independent variables are provided in Table 3.

**Table 3:** Means, standard deviations and bivariate correlations (Spearman Rho) of demographic and self-report variables and conjoint relative importance scores (*n* = 586).

		**Mean**	**SD**	**1**	**2**	**3**	**4**	**5**	**6**	**7**	**8**	**9**	**10**	**11**	**12**	**13**	**14**
1	Gender (0 = men; 1 = women)	0.68	–	1.00													
2	Age (Winsorized to 32 years) (*n* = 583)	23.77	3.24	−0.09^*^	1.00												
3	International (1 = yes)	0.20	–	−0.16^**^	0.17^**^	1.00											
4	Engineering (1 = yes)	0.10	–	−0.36^**^	−0.05	0.14^**^	1.00										
5	Entrepreneurship (1 = yes)	0.11	–	−0.15^**^	0.10^*^	0.52^**^	−0.12^**^	1.00									
6	Nursing (1 = yes)	0.30	–	0.32^**^	−0.33^**^	−0.21^**^	−0.22^**^	−0.23^*^	1.00								
7	Science (1 = yes)	0.10	–	−0.19^**^	−0.15^**^	0.02	−0.11^**^	−0.12^**^	−0.21^**^	1.00							
8	Veterinary science (ALL)	0.40	–	0.13^**^	0.36^**^	−0.23^**^	−0.27^**^	−0.29^**^	−0.52^**^	−0.26^**^	1.00						
9	Veterinary science 1 (1 = yes)	0.14	–	0.06	0.25^**^	−0.12^**^	−0.14^**^	−0.15^**^	−0.26^**^	−0.13^**^	0.51^**^	1.00					
10	Veterinary science 2 (1 = yes)	0.17	–	0.07	0.14^**^	−0.22^**^	−0.15^**^	−0.16^**^	−0.29^**^	−0.15^**^	0.55^**^	−0.18^**^	1.00				
11	Veterinary science 3 (1 = yes)	0.09	–	0.07	0.13^**^	0.03	−0.10^*^	−0.11^**^	−0.20^**^	−0.10^*^	0.38^**^	−0.13^**^	−0.14^*^	1.00			
12	Prosociality (1–7)	4.28	0.95	0.20^**^	−0.09^*^	0.10^*^	−0.03	0.00	0.30^**^	−0.15^**^	−0.17^**^	−0.10^*^	−0.08^*^	−0.05	1.00		
13	Income importance career (1–7)	5.17	0.94	−0.15^**^	0.03	0.21^**^	0.12^**^	0.16^*^	0.02	−0.05	−0.16^**^	−0.08	−0.13^**^	−0.02	−0.03	1.00	
14	Other Orientation	0.21	0.25	0.13^**^	−0.04	−0.05	−0.10^*^	−0.05	0.18^**^	0.02	−0.09^*^	0.07	−0.16^**^	−0.03	0.32^**^	−0.24^**^	1.00
15	Self-Interest	0.65	0.30	−0.14^**^	0.03	0.01	0.05	0.05	−0.22^**^	0.04	0.12^*^	−0.03	0.10^*^	0.12^**^	−0.36^**^	0.23^**^	−0.69^**^

* Correlation is significant at the 0.05 level (2-tailed).

** Correlation is significant at the 0.01 level (2-tailed).

The data provided in Table 3 shows that overall, our study population was positive for other-orientation (*u* = 0.21) and self-interest (*u* = 0.65). Correlations between observed items, composite forms of self-report Likert scale measures, and the part-worth utilities from the metric conjoint experiment appeared reasonable. Other-orientation correlated negatively with self-interest (*r* = −0.69, *p* < 0.01) and income importance (*r* = −0.24, *p* < 0.01) and positively with prosociality (*r* = 0.32, *p* < 0.01). Self-interest correlated negatively with prosociality (*r* = −0.36, *p* < 0.01), positively with Income Importance (*r* = 0.23, *p* < 0.01), and had no other notable correlations negative or positive except a negative correlation with being a nursing respondent (*r* = −0.22, *p* < 0.01).

### Other-orientation and self-interest of respondents per discipline and gender (*n* = 586)

**Figure 2:**
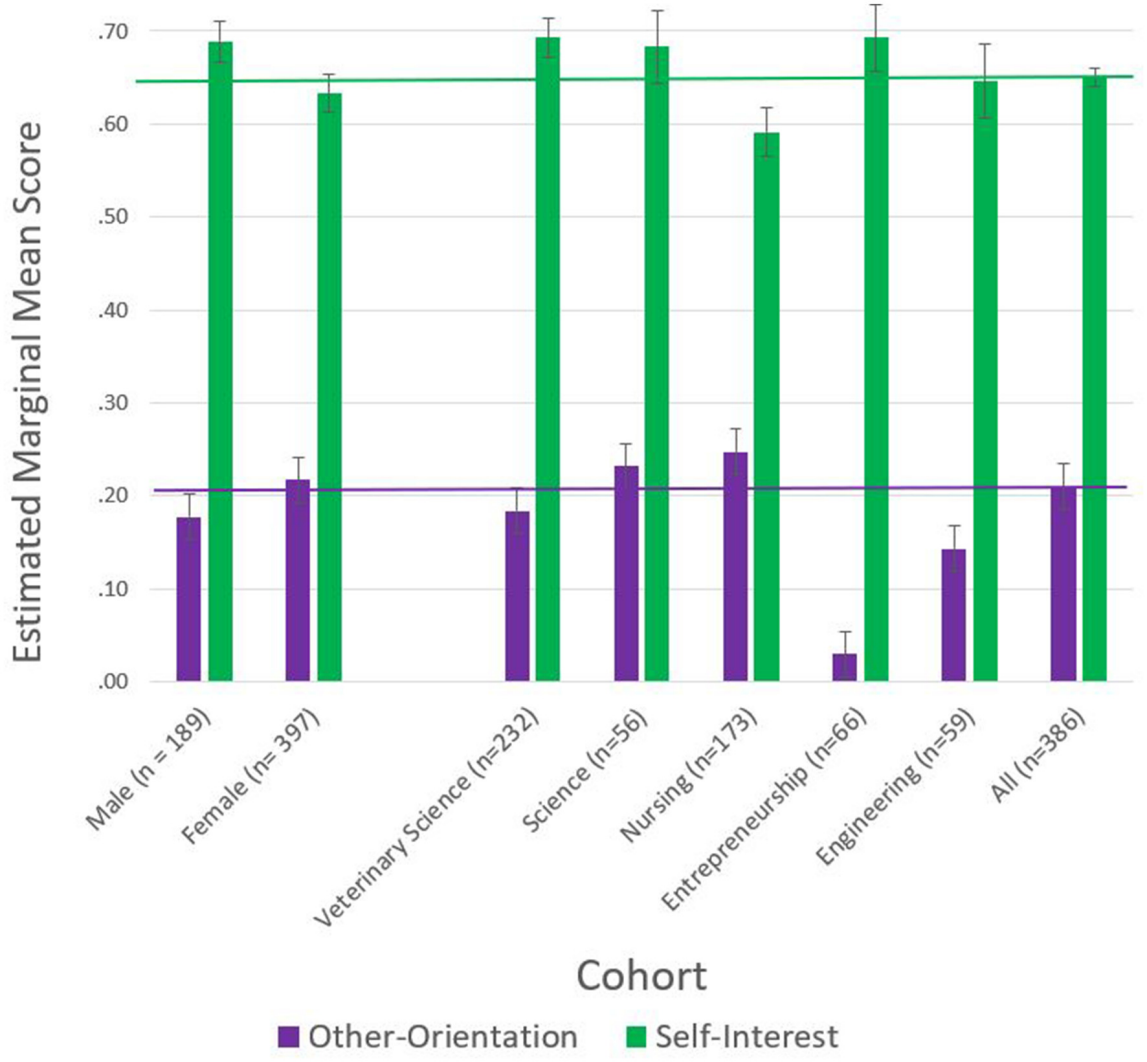
Estimated marginal means from the MANOVA of other-orientation and self-interest per discipline and gender (*n* = 586). Horizontal lines represent study population means for “other-orientation” (*u* = 0.21) and “self-interest” (*u* = 0.65).

We undertook a two-way multivariate analysis of variance (MANOVA) of other-orientation and self-interest per discipline and gender to address *Hypotheses 1a, 1b, 2a and 2b*. We provide the MANOVA results in Table 4, Figure 2, and Supplementary Tables 3, 4. In Table 4, small *p*-values of a test statistic (*p* < 0.05) mean the test has returned a significant result and that there is a difference between levels of the independent variable on the dependent variable. The partial ETA squared figures in the right-hand column can be viewed as the “percentage of variance” by multiplying by 100. For example, discipline significantly contributes to the dependent variable for other-orientation with an estimated “Partial ETA squared” of 0.018 or 1.8% and Self-Interest with an estimated “Partial ETA squared” of 0.022 or 2.2%.

**Table 4:** Two-way MANOVA Tests of Between-Subjects Effects on other-orientation and self-interest simultaneously (*n* = 586).

**Source**	**Dependent variable**	**Type III sum of squares**	**df**	**Mean square**	***F***	**Sig**.	**Partial eta squared**
Corrected model	Other-orientation	1.160^a^	5	0.232	3.794	0.002	0.032
	Self-interest	1.858^b^	5	0.372	4.293	<0.001	0.036
Intercept	Other-orientation	16.008	1	16.008	261.918	<0.001	0.311
	Self-interest	180.429	1	180.429	2,083.953	<0.001	0.782
Gender	Other-orientation	0.149	1	0.149	2.444	0.119	0.004
	Self-interest	0.303	1	0.303	3.498	0.062	0.006
Discipline	Other-orientation	0.644	4	0.161	2.635	0.033	0.018
	Self-interest	1.127	4	0.282	3.255	0.012	0.022
Error	Other-orientation	35.450	580	0.061			
	Self-interest	50.216	580	0.087			
Total	Other-orientation	62.302	586				
	Self-interest	297.660	586				
Corrected total	Other-orientation	36.609	585				
	Self-interest	52.075	585				

a R Squared = 0.032 (Adjusted R Squared = 0.023).

b R Squared = 0.036 (Adjusted R Squared = 0.027).

The data in Table 4 shows the MANOVA outcomes. The variance explained for self-interest in the MANOVA model was greater than in its ANOVA. In the MANOVA, the interaction term (two-way) between gender and discipline decreased the variance explained, so we removed it from the model. Overall, the variance explained in each dependent variable by the independent demographic variables gender and discipline was very small in the MANOVA model.

Supplementary Table 3 provides the estimated marginal means of each dependent variable, other-orientation and self-interest, per gender and discipline derived from the MANOVA. Supplementary Table 4 provides the pairwise comparisons of the means of the dependent variables per gender and discipline from the simultaneous MANOVA of other-orientation and self-interest. Figure 2 illustrates the estimated marginal means of other-orientation and self-interest per discipline and per gender.

Figure 2 and Supplementary Tables 3, 4 show that when ranked, engineering respondents were the least other-oriented, and nursing respondents were the most other-oriented. Nursing respondents were the least self-interested, while entrepreneurship and veterinary respondents were the most self-interested.

Veterinary respondents were less other-oriented than nursing respondents (*p* = 0.021), thus not supporting *Hypothesis 1a*. Veterinary respondents were more self-interested than nursing respondents (*p* = 0.002), thus not supporting *Hypothesis 1b*.

While male respondents ranked lower in other-orientation and higher in self-interest than women respondents, pairwise comparisons, multivariate tests and univariate tests showed no differences (*p* > 0.05). Therefore, there is no support for *Hypothesis 2a* and *Hypothesis 2b* from the MANOVA procedure.

### Other-orientation and self-interest of veterinary respondents (*n* = 232)

We undertook a multivariate analysis of variance (MANOVA) of other-orientation and self-interest for gender for the veterinary sub-population (*n* = 232) to address *Hypotheses 3a and 3b* (see Table 5).

**Table 5:** One-way MANOVA tests of between-subjects effects on other-orientation and self-interest simultaneously of the veterinary sub-population (*n* = 232).

**Source**	**Dependent variable**	**Type III sum of squares**	**df**	**Mean square**	***F***	**Sig**.	**Partial eta squared**
Corrected model	Other-orientation	0.023^a^	1	0.023	0.314	0.576	0.001
	Self-interest	0.124^b^	1	0.124	1.377	0.242	0.006
Intercept	Other-orientation	6.073	1	6.073	84.189	<0.001	0.268
	Self-interest	82.464	1	82.464	918.246	<0.001	0.800
Gender	Other-orientation	0.023	1	0.023	0.314	0.576	0.001
	Self-interest	0.124	1	0.124	1.377	0.242	0.006
Error	Other-orientation	16.592	230	0.072			
	Self-interest	20.655	230	0.090			
	Other-orientation	25.324	232				
	Self-interest	127.683	232				
Corrected total	Other-orientation	16.615	231				
	Self-interest	20.779	231				

a R Squared = 0.001 (Adjusted R Squared = −0.003).

b R Squared = 0.006 (Adjusted R Squared = 0.002).

The estimated marginal means of other-orientation of men veterinary respondents (*u* = 0.18, s.e. 0.04) did not differ (*p* = 0.58) from that of women veterinary respondents (*u* = 0.20, s.e. 0.02). Therefore, *Hypothesis 3a* was supported.

The estimated marginal means of self-interest of men veterinary respondents (*u* = 0.72; s.e. 0.04) did not differ (*p* = 0.24) from that of women veterinary respondents *(u* = 0.67; s.e. 0.02). Therefore, *Hypothesis 3b* was not supported.

### *Post-hoc* hierarchical multivariate linear regression, including additional demographic and attitudinal effects on other-orientation and self-interest

To add to our understanding of the role of discipline and gender on other-orientation and self-interest of our study respondents, we undertook a *post-hoc* multivariate hierarchical linear regression, with other-orientation and self-interest as the dependent variables (see Supplementary Table 5). The data in Supplementary Table 5 shows the variance explained in the dependent variables directly by demographic variables was similar for other-orientation (6.1%) and self-interest (6.3%; *Model 1*). Adding interaction terms for the demographic variables explained 0.6% of other-orientation and 0.7% of self-interest (*Model 2*). The addition of the composite variables, prosociality and income importance, explained an additional 12.7% of other-orientation and 7.7% of self-interest (*Model 3*).

In summary, for our study population and relevant to our hypotheses, after incorporating other relevant demographic and attitudinal variables:

- Gender did not affect other-orientation directly. International student status had a medium effect on other-orientation. However, together, gender and international student status had a small-medium positive effect on other-orientation. Gender had a strong negative affect on self-concern, which in a two-way interaction with age, was amplified.
- Being a nursing, science, or veterinary respondent (compared to being an engineering respondent) positively affected other-orientation, but university 2 related factors can negate this for a veterinary individual.
- Discipline did not affect the level of self-interest in an individual, however, institution-related factors (being a veterinary respondent attending university 2 or university 3) positively affected self-interest.

### Distinguishable groups of respondents for cluster attributes of other-orientation and self-interest

To address *Hypothesis 4*, we undertook a *K*-means cluster analysis of other-orientation and self-interest for the study population (*n* = 586). The clustering analysis revealed four interpretable groups, with the smallest clusters >5% of the study population and the largest cluster at 48.6% of the study population. After classification into the four clusters, we performed a multivariate analysis of variance (MANOVA) for the cluster attributes other-orientation and self-interest across the four clusters. The MANOVA demonstrated statistically significant differences between clusters for Other-Orientation [*F*_(3, 582)_ = 547.236, *p* < 0.001] and for self-interest [*F*_(3, 582)_ = 739.729, *p* < 0.001]. Other-Orientation scores differed between all clusters (*p* < 0.001; Games Howell). Self-Interest scores were not different between cluster 2 and cluster 4 but differed between the other clusters (*p* < 0.001; Games Howell).

**Figure 3:**
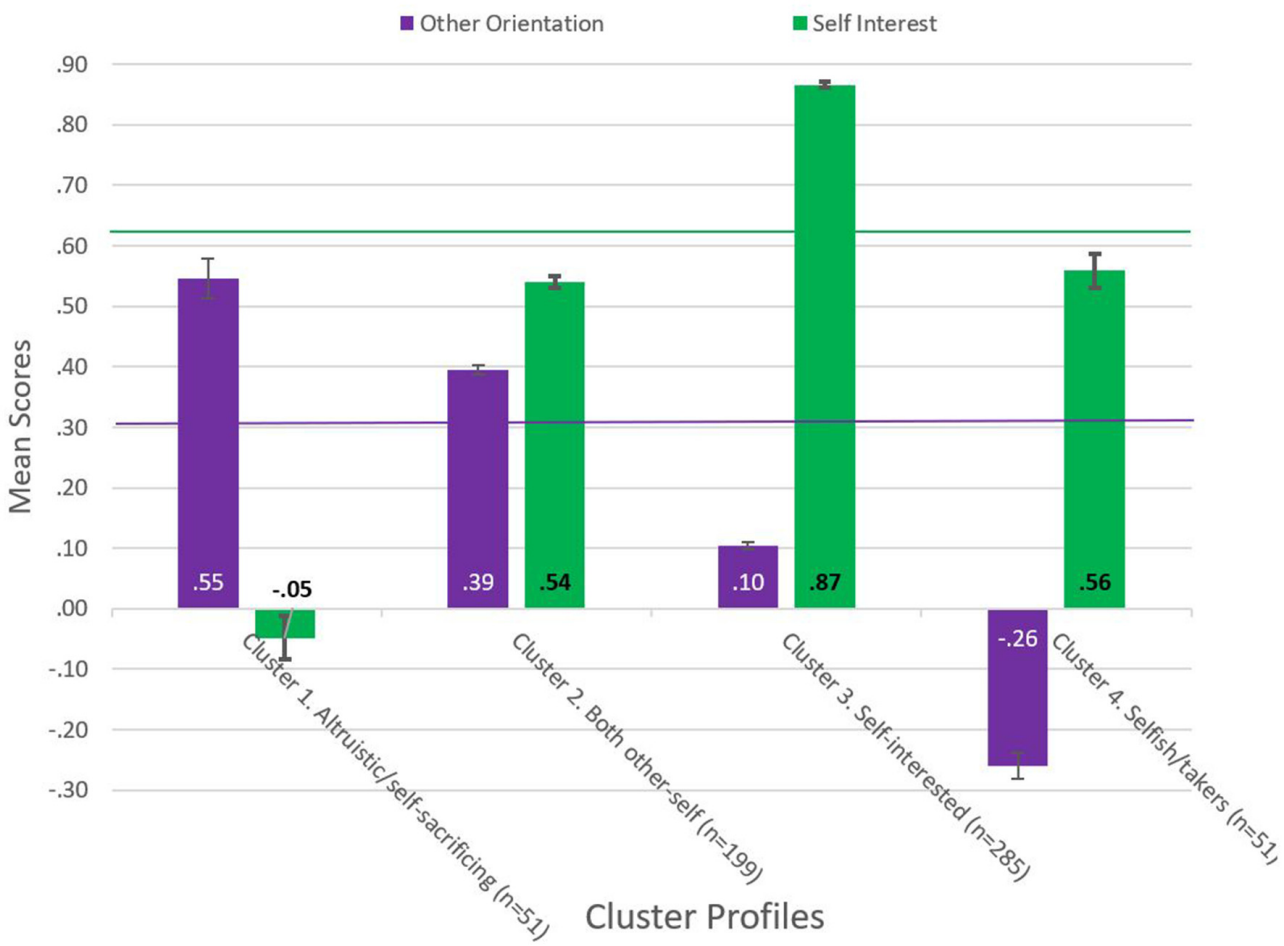
Mean scores (s.e.) of other-orientation and self-interest to respondents in the four cluster profiles (*n* = 586). Horizontal lines indicate the sample population mean for other-orientation (*u* = 0.21) and self-interest (*u* = 0.65).

Figure 3 and Supplementary Table 6 show the means of Other-Orientation (OO) and Self-Interest (SI) for each cluster profile arising from the *K*-means clustering analysis.

The first group (*n* = 51, 8.7%) to emerge from the cluster analysis (Cluster 1 = High OO Neg SI) identified respondents with the highest scores (well above the mean) for other-orientation coupled with slightly negative scores for self-interest. We labeled this profile as “altruistic/self-sacrificing.”

The second group (*n* = 199, 34.0%) to emerge from the cluster analysis (Cluster 2 = High OO Med SI) identified respondents with scores above the mean for other-orientation, and below the mean but still positive for self-interest. We labeled this profile “both other-self.”

The third and largest group (*n* = 285, 48.6%) to emerge from the cluster analysis (Cluster 3 = Low OO High SI) identified respondents with scores for other-orientation well below the mean. We labeled this profile as “self-interested.”

The fourth group (*n* = 51, 8.7%) to emerge from the cluster analysis (Cluster 4 = Negative OO Med SI) identified respondents who, in contrast to the other three clusters, had a negative level of other-orientation (i.e., an aversion to helping others) coupled with positive self-interest (though below the mean). We labeled this profile as “selfish-takers.”

For naming our cluster profiles, we have leaned toward the terminology used in the literature (16, 108). The distinctness of the four interpretable cluster profiles provides support for *Hypothesis 4*.

### Representation of cluster profiles within discipline

**Figure 4:**
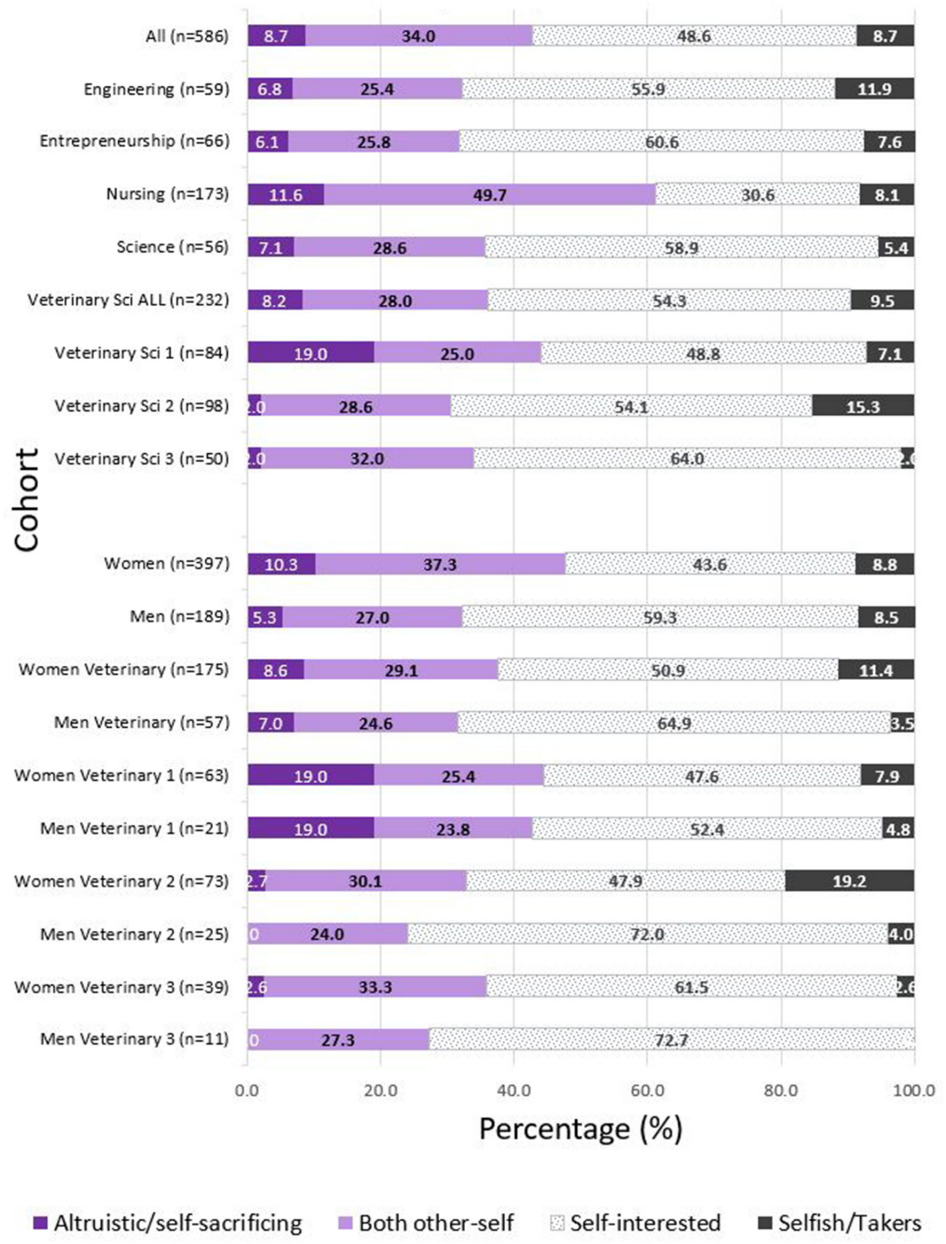
Proportional representation of the four clusters for the disciplinary cohorts of the study population (*n* = 586).

To address *Hypotheses 5*, we undertook a frequency analysis of the cluster profile memberships for each disciplinary cohort (see Figure 4).

Figure 4 provides the proportions of each cluster profile per disciplinary cohort. “Altruistic/self-sacrificing” (Cluster 1) respondents were highly represented in veterinary science (8.2%) and nursing (11.6%). “Both other-self” (Cluster 2) respondents were most highly represented in nursing (49.7%) and then veterinary science (28.0%). “Self-interested” (Cluster 3) respondents were most highly represented in science (60.9%) and entrepreneurship (60.6%). “Selfish/Takers” (Cluster 4) respondents were most highly represented in veterinary science (9.5%) and engineering (11.9%). Overall, the diversity in the proportional representation of the cluster profiles of veterinary and other disciplinary cohorts provided support for *Hypothesis 5*.

### Representation of cluster profiles within veterinary program

To address *Hypotheses 6* we undertook a frequency analysis of the cluster profile memberships for the three participating veterinary programs (see Figure 4).

Notably, the proportional representation of the cluster profiles across the three veterinary cohorts greatly differed, providing support for *Hypothesis 6*.

Figure 4 also illustrates the proportions of cluster memberships for all male and female respondents of our study population and women and male veterinary respondents of the three participating veterinary schools. Overall, membership of Cluster 4 (“selfish/takers”) was similar for women (8.8%) and men (8.5%), but membership of Cluster 3 (“self-interest”) was lower for women (43.6%) than men (59.3%). Correspondingly, membership in Cluster 2 (“both other-self”) and Cluster 1(“altruistic/self-sacrificing”) was higher for women (37.3%; 10.3%) than for men (27.0%; 5.3%). Women and men veterinary respondents in the different veterinary schools (except men of veterinary science 3) were overall similar in membership of Cluster 2 (“both other-self”) (24.0–33.0%). Veterinary science 2 women respondents stood out for 19.0% membership of Cluster 4 (“selfish/takers”). In comparison, veterinary science 1 women and men respondents stood out for both being high in membership of Cluster 1 (“altruistic/self-sacrificing”; both 19.0%).

Thus, four distinct and interpretable cluster-based profiles emerged for the sample population based on other-orientation and self-interest. In particular, the most notable features illustrated in Figure 4 are two-fold:

- The high proportion of altruistic and caring attributes in respondents in studying nursing and veterinary science at university 1.
- The difference in the cluster memberships of entrants to the veterinary profession associated with their veterinary school.

## Discussion

In this research, we approach other-orientation as a relatively stable value-driven personality trait (109) that a socialized “collective identity” may also reinforce. Motivated by the Theory of Other Orientation and related behavioral science and known wellbeing issues in the veterinary profession (23, 24), a “caring and patient-focused” occupation, we took the first step in ascertaining the extent of other-orientation and self-interest as traits of tomorrow's veterinarians. We designed a metric conjoint experiment, drawing on decision rule and microeconomic utility theories (91, 92, 110) to test our hypotheses. We used data from 586 experimental surveys answered by our respondents, who were soon to enter veterinary, human nursing, and three non-patient centered tertiary-qualified occupational workforces. Respondents' other-orientation was determined in a choice scenario involving trade-offs when considering job profiles. We used the metric conjoint experiment part worth outputs to reveal (as opposed to self-report) respondents' other-orientation relative to their self-interest. We investigated and explored a number of hypotheses regarding the levels of other-orientation and self-interest alone and in combination, for our study population, chosen for its likely variance.

In contrast to other scholars (61, 62, 65, 66), we found, empirically and experimentally, that being female was not associated with greater other-orientation, and being male was not associated with greater self-interest, in our study population using multivariate analysis of both discipline and gender. When we tested and retained other variables in our *post-hoc* multivariate linear regression model, any effects of gender on other-orientation fell away. That is, gender accounted for the other predictor variables before their inclusion. We believe that demographic variables, including gender, act as “proxies” for otherwise unmeasured human capital (e.g., International student status), social norms/collective identity beliefs (university, income importance) and personal values (e.g., prosociality).

Whether women are more other-oriented than men will depend on context, personal cost, and other constructs that cannot be accounted for practically in every study (32, 64). That in our comparison of women and men veterinary respondents, we found no difference in other-orientation, was not surprising. The main motivation to study or practice veterinary medicine is to help and care for “animals,” likely to be driven by empathic concern irrespective of one's gender. We also found no gender difference in self-interest for veterinary respondents. Our self-interest measure, formed by combining the relative importance of income and the relative importance of work effort, may have masked a gender difference in self-interest related to remuneration levels. However, we would hope our findings indicate a narrowing of any gender gap in pay expectations for our study population, given most participants residing in a western educated, industrialized, rich and democratic (WEIRD) country (34).

That an altruistic/self-sacrificing cluster profile emerged in which respondents were highly other-oriented coupled with sub-zero self-interest is perhaps a more useful indicator of the willingness of respondents, male or female, to disregard opportunity for personal reward to facilitate their helping behavior (for their clients and patients benefit). The clusters that emerged for our study population corroborate the conjecture in the public economics literature (10) and the medical social sciences literature (9) that other-regarding motivation is closely associated with the personal sacrifices a professional provider would be willing to make and the context. When undertaking the metric conjoint experiment, participants were asked to consider the scenarios in the context of their disciplines. The role of context is seen when overall, women were nearly twice as frequent members of the altruistic/self-sacrificing cluster than men, but for veterinary respondents, the percentage of men and women in the “altruistic/self-sacrificing” profile was more similar, particularly for veterinary respondents of the university 1.

Had we not included veterinary respondents from two other Australian veterinary schools, this study would have suggested that veterinary respondents could be considered of greater other-orientation than respondents from different disciplines. That being from the veterinary programs of university 2 or 3 was associated with lower other-orientation and increased self-interest, may reflect factors associated with the respective veterinary school, e.g., admission process (one of the veterinary schools selects for rural background), school culture and related social norms, human capital, and “collective identity” vicarious learning/norming while training. The underlying context is likely related to veterinary school differences. For example, the admissions processes of these veterinary schools differ from the academic merit-based entry of university 1 (as the time of our survey). While differing societal norms or collective identity effects may contribute to the differences between veterinary respondents of the three veterinary schools in their cluster membership, the length of veterinary study programs (5–6 years) compared to the 3 or 4-year programs of the other disciplines, may well further contribute (75).

Four distinguishable cluster profiles emerged for our study population for the attributes of other-orientation and self-interest that were interpretable with respect to theory and terminology in the health economics and behavioral sciences literature. For example: “knights” (cluster 1) and “knaves” (cluster 3) (56), “other-oriented” (cluster 1) and “self-concerned” (cluster 3, cluster 4) (11, 26, 35), “givers” (cluster 1, cluster 2) and “takers” (cluster 4) (25) or the social motive profiles' self-sacrifice (cluster 1), both-oriented (cluster 2), and self-interest (cluster 3, cluster 4)' (16, 108). The frequency analysis showing the proportions of each cluster per occupational cohort was most interesting. Specifically, one veterinary school (university 1) had a greater proportion of respondents in the altruistic/self-sacrificing cluster profile than nursing. Conversely, this same veterinary school cohort had the smallest proportion of respondents in the “both-other-self” cluster profile. This descriptive finding further corroborates the conjecture in the health economics literature (10) that other-regarding motivation is closely associated with the personal sacrifice the professional provider would be willing to make.

### Contributions

Our study contributes to our understanding of veterinary other-orientation and self-interest in four ways. First, we summarize relevant literature to argue why it is important to understand that other-orientation and self-interest would be diversely represented across any population, particularly the veterinary population. Such understanding is necessary to craft appropriate organizational support for veterinary clinicians at the client interface.

Second, we described and illustrated how other-orientation and self-interest are present empirically in a sample of prospective veterinarians. We did this using a metric conjoint experiment rather than self-reported data. By forcing trade-off decision-making behavior, we mitigated against acquiescence bias for the variables generated from the conjoint analysis of the experiment. The extant veterinary social science literature has not reported this procedure and particular analysis.

Third, we demonstrate that the metric conjoint experiment can be used as a tool for scholars undertaking quantitative veterinary social science studies. Choice experiments are not common in organizational science or medical education research. Yet, within the context of our study, the metric conjoint experiment constitutes a valid research method for critically assessing personal attitudes and normative beliefs by involving trade-off decision-making. We believe that the metric conjoint experiment allows discernment of “both other-self” from “altruistic/self-sacrificing” other-orientation. The conjoint experiment and analysis enable discrimination and robustness to measure other-orientation in various combinations with self-interested dispositions for our context.

Fourth, we responded to the call of Coulter et al. (6) for further study of the gender difference in altruistic attitudes of medical sector entrants. Coulter et al. (6) conjectured that if other studies supported the gender difference observed in their research, it might foreshadow a considerable change in medical practice as the percentage of female medical students increases across the USA and Europe. Our findings indicate that the values and hidden curriculum of a medical (or veterinary) school may be the more influential factor on the level of other-orientation compared to self-interest and not the gender of the prospective medical (or veterinary) practitioner. That is, admissions processes and collective identity factors are likely more involved than gender, *per se*.

### Limitations

There are some limitations associated with this study. *First*, it is cross-sectional. A study design involving multiple measurement points would have been ideal. Time, financial, and other resource limitations prohibited us from doing this. *Second*, while decreasing reliance on self-reporting, the conjoint paper-based environment utilized in this research is not equivalent to a real-world setting. *Third*, we diverted from the recommended duplicate orthogonal array of hypothetical job positions (85) for the 2016 and 2017 data collections in response to participants in the year 2015 commenting negatively about having to rate the same job scenario twice and having to do the paper survey over two classes. Thus, we traded off the ability to fully test-retest all eight hypothetical job scenarios as a validity check of the conjoint experiment, to only test-retest one job scenario, to minimize disengagement and imposition on participants. Our decision was supported by Pearson correlations (*r* > 0.6) between the duplicated responses and evidence of engagement with the survey instrument in the open-ended text questions included after various banks of survey items. *Fourth*, participants might be considered inherently more prosocial than non-participants; we believe we mitigated this by running the experiment and survey in class, explaining the study's relevance directly to participants, and offering non-monetary rewards such as chocolates and cakes. *Fifth*, the study was limited by only sampling one university for non-veterinary participants. The sampling of veterinary students from multiple institutions was begun in 2015, when preliminary data analysis revealed that restriction of the sample population to veterinary respondents could be an issue, as variance in key variables of interest could be too narrow, impacting the theoretically informed modeling to be performed on the data. Additional ethics and participants were able to be quickly obtained for the primary institution but was too large an imposition on non-veterinary students and unknown colleagues in universities 2 and 3. The non-veterinary respondents of university 1 were chosen on the basis of (i) heterogeneity in personal values in relation to graduate career choices (33); and (ii) some shared characteristics with the veterinary discipline i.e., science and technology (engineering and science), service delivery *via* private enterprise (entrepreneurship/business students) and delivery of patient centered medical care (nursing). *Sixth*, the samples of engineering, entrepreneurship and science programs were relatively small, compared to the nursing respondents sample. The participation rate of the nursing sample was high, associated with their excellent attendance rates, compared to the other programs, and a black out occurring just prior to the second entrepreneurship class participation. For this study, exclusion of non-veterinary respondents would have denied the reader the opportunity to “benchmark” our findings with other patient-focused and non-patient focused respondents. This decision was supported by veterinary audiences at two conference presentations, who were highly interested in the frequency analysis of the cluster profiles of veterinary respondents compared to the other cohorts. On the other hand, exclusion of university 2 and 3 veterinary cases from this study, given the study population's lack of non-veterinary responses from these institutions, would have resulted in a missed opportunity to expose the effect of learning institution on traits such as other-orientation and self-interest. The differences in other-orientation and self-interest for veterinary respondents of university 2 and 3 to the primary institution highlights the need for need for more research to properly understand if origins are in admissions/selection, hidden/taught curriculum or other factors.

### Implications for future research

Our study has implications for researchers in the other-orientation/prosociality/rational self-interest space. Four discernible and interpretable clusters emerged from our study population responses to our metric conjoint experiment, which made theoretical and common sense (15, 16, 25, 35, 108). Our results may encourage other researchers to use the metric conjoint experiment to add validity and depth to future related studies.

In particular, and as discussed abover, the attributes of our cluster profiles align with the literature. For example, our “altruistic/self-sacrificing” profile supports altruistic other-orientation as a separate dimension to other forms of prosociality (111); our “selfish/taker” profile endorses the existence of a “taker” persona (25), distinct to just a “self-interested” profile; our “both other-self” profile aligns with the position that people may be both other-oriented and self-interested at the same time (16, 35, 108) and this is important on two counts. First, it fits with the literature that although other-orientation and self-interest are separate constructs. Second, that being able to be both, has been linked with sustainability and wellbeing in professional contexts requiring repeated other-oriented behaviors in individuals (16).

Joining practice to theory, the other-orientation scholar could consider the veterinary context in which, with no underpinning national health insurance schemes for animal care, the pressure for altruistic behavior vs. the practical need for reward (physical, mental, or financial) presents an ongoing tension for veterinary team members and business owners. Our findings of the presence of an “altruistic/self-sacrificing” profile in the veterinary context may stimulate interest in further research to address the relationship between personality values and traits, organizational behavior constructs, and the morbidity and mortality issues associated with burnout and compassion fatigue in caring professions. On a positive note, based on the link made by Bolino and Grant (16) with sustainability and wellbeing, research linking the “both other-self” profile with job satisfaction and intention to stay measures, to establish if interventions to increase representation in this profile type would be worthwhile.

### Implications for policymakers, educators, and veterinary schools

The new knowledge from our study has implications regarding outcomes from admissions processes and curricula' potential (or dysfunction) for supporting student and graduate psychological and financial wellbeing development. Our finding that the cluster distribution differed markedly among the three veterinary schools has implications for discipline-based policymakers and veterinary educators. We believe that these differences may be consequential to (1) the three participating veterinary schools having differing admissions procedures in place at entry level, (2) self-selection playing a role in the values and behavior of the incumbents, and (3) vicarious alignment of respondents with the collective identity of their veterinary school noting that the values of a school can affect the values of its graduands and graduates (75). Cohorts may be impacted by personality and value differences in applicants and motivational predispositions at entry (112) in addition to ability and achievement measures required by (health-sciences) admissions processes (113–115). One might argue that educational influences may also influence proportional profile differences for different cohorts (vicarious or planned “indoctrination”) (9). Like Hennig-Schmidt and Wiesen (9), our results do not imply that individuals who choose non-patient focused disciplines start or finish more self-interested than those who choose patient-caring focused disciplines such as nursing or veterinary.

The results of this study have implications for professional program admission committees. Some health science disciplines (e.g., dentistry) recognize that it would be advantageous to have more applicants willing to work in underserved communities (76). In contrast, other disciplines may wish to graduate fewer “altruistic/self-sacrificing” types to partly address their profession's problem with burnout, compassion fatigue, and suicide. Many dental, medical, and veterinary schools include non-cognitive and cognitive criteria in multiple mini-interview (MMI) stations (116) and or Situational Judgement Test (SJT) scenarios to discern values-based attributes in applicants and even help screen for applicants of high integrity and “moral” orientation (117, 118).

### Implications for the veterinary profession

There are several practical implications arising from our study for the veterinary profession. First, by illustrating the diversity in other-orientation and self-interest configurations in soon-to-graduate veterinarians, we provide a new lens for employers and policymakers looking to implement post-graduate educational initiatives to support the psychological and financial wellbeing of members of the veterinary profession and organizational performance.

Second, although other-oriented (or prosocial) motives and behaviors are intended to benefit others, they may have negative consequences, including being easily pressured or obligated to help and sacrifice their energy and effectiveness (16). Risk factors for anxiety, burnout, compassion fatigue, depression etc., are associated with other-orientation, particularly when not coupled with sufficient self-concern (111). Being an altruistic-self-sacrificing type could be detrimental without appropriate discernment and judgement (25, 110); individuals may over or under-respond in their day-to-day work, leading them to apathy, burn out or compassion fatigue (13, 21). Development of skills in discernment and judgement becomes even more important, if not critical, for those in roles demanding high levels of other-centeredness and altruism (13, 21). As such, the findings of our study provide a platform for those who seek to address the tension in veterinary service provision where daily demand or perceived demand for altruistic and other-oriented service provision is a constant pressure.

On the other hand, researchers in the organizational sciences continue to identify how other-orientation or prosociality (concern for the welfare of others) can enhance individual and organizational effectiveness (16, 25). The Theory of Other-Orientation alerts us that other-oriented individuals differ from more self-interested individuals, in their valences and decision-making processes, even in non-helping situations, with associated pros and some cons for organizational citizenship behaviors, self-appraisal, and even the relationship between chief executive officer characteristics and organizational performance (15). Veterinary employers and managers interested in job satisfaction, organizational citizenship behaviors, productivity, and retention, should know that differing other-orientation affects the priorities and attitudes of members of their veterinary teams. Our finding of the “both other-self” cluster profile present in 25–32% of veterinary respondents compared to 49.7% of nursing respondents has implications for practice. It is acknowledged that high prosocial motivation coupled with high self-concern appears to generate the most sustainable contributions to others [(119, 120) per (16)]. More veterinary respondents in this cluster would probably benefit individual wellbeing, team functioning and therefore sustainability of the profession at the individual and organizational level.

## Conclusions

The metric conjoint experiment is an experimental measurement instrument useful for revealing other-orientation and self-interest in individuals. This tool may interest researchers looking to avoid reliance on “self-reporting” using Likert-like response formats.

Other-orientation and self-interest can be represented in a sub-population (such as our study population) as one of four typical combinations or cluster “persona” profiles: altruistic/self-sacrificing, primarily self-interested, selfish/takers, and as both “other-oriented/self-interested.” The latter profile is considered important for organizational and personal sustainability and wellbeing, and more veterinary respondents in this cluster could enhance sustainability of the profession at the individual and organizational level.

Altruistic/self-sacrificing other-orientation can be more represented in some veterinary school cohorts and less so in others, raising questions about the role of admissions processes and collective identity' acquisition in hidden or taught veterinary curricula.

## Data availability statement

The raw data supporting the conclusions of this article will be made available by the authors, without undue reservation.

## Ethics statement

The studies involving human participants were reviewed and approved by University of Adelaide HREC H-2015-114. Written informed consent for participation was not required for this study in accordance with the national legislation and the institutional requirements.

## Author contributions

AF: conceptualization, methodology, formal analysis, data curation, software, validation, writing—original draft preparation, writing—review and editing, project administration, funding acquisition, resources, and visualization. NL: conceptualization, writing—review and editing, funding acquisition, and visualization. PS: conceptualization, data curation, methodology, validation, visualization, and writing—review and editing. EP: conceptualization, data curation, writing—review and editing, and visualization. All authors contributed to the article and approved the submitted version.

## Footnotes

^1^Skill variety, task identity, task significance, autonomy, and feedback (28).

^2^The theory of reasoned action, and the theory of planned behavior (31, 38, 39).

^3^Thus, other-orientation involves more than the pursuit of other-oriented goals. It also involves less reliance on systematic processing in the pursuit of one's goals [see (12, 29, 41)].

^4^The “right dorsolateral pre-frontal cortex” (“rDLPFC”).

^5^Similarly, age could potentially affect other-orientated decision heuristics in trade-off scenarios as increasing age can represent the increased acquisition of human capital (social and physical assets). Further, nationality can be a “catch-all” proxy for family background and social norm differences between individuals (e.g., international tertiary students) (63).

^6^Shared beliefs about what is expected and what is admirable of individuals related to their sex.

^7^Attending the same university as the majority of our respondents.

^8^This time horizon was used because we argue that 12–24 months appears to be sufficient time for a respondent to prepare for a career change, while we considered that graduates' main consideration for their first job might be to get experience as quickly as possible.

^9^SC_RI_ = INC_RI_-WK_RI_ = [(INC_pw2_-INC_pw1_)–(WK_pw2_-WK_pw1_)]/[abs(OO_pw2_-OO_pw1_) + abs(INC_pw2_-INC_pw1_) + abs(WK_pw2_-WK_pw1_)].

^10^Fit metrics were not reported for the scale by Coulter et al. (6) or by Xu et al. (63); therefore the scale may also have behaved similarly for their study populations.

^11^We used the more sensitive *p* > 0.001 due to our study population being > 200 (104, 107).
