# Supplementary material for: Altruism or self-interest in tomorrow's veterinarians? A metric conjoint experiment and cluster analysis
Source: Front Vet Sci. 2023 Apr 5;10:1044463. doi: 10.3389/fvets.2023.1044463 (PMC10113617; doi:10.3389/fvets.2023.1044463)
Supplement: Supplementary file 1 [file Data_Sheet_1.docx]

**Supplementary TABLE 1.** Demographic profile of respondents before data hygiene (n = 639).

|  | Total | Median age | International % (n) | Male% (n) | Female % (n) |
| --- | --- | --- | --- | --- | --- |
| Engineering 1 | 62 | 22.0 | 37.1% (n=23) | 82.3% (n=51) | 17.7% (n=11) |
| Entrepreneurship 1 | 73 | 24.0 | 76.7% (n=56) | 56.2% (n=41) | 43.8% (n=32) |
| Nursing 1 | 198 | 21.0 (n=195) | 6.6% (n = 13) | 9.1% (n=18) | 89.9% (n=178) |
| Science 1 | 63 | 22.0 | 25.4% (n=16) | 57.1% (n=36) | 41.3% (n=26) |
| Veterinary Science (ALL) | 243 | 24.0 (n=241) | 8.6% (n=21) | 23.9% (n=58) | 76.1% (n=185) |
| **Total** | **639** | **23.0^ (n=634)** | **20.2% (n=129)** | **31.9% (n=204)** | **67.9% (n=432)** |
|  |  |  |  |  |  |
| Veterinary Science 1 | 90 | 24.0 (n=89) | 7.8% (n=7) | 23.3% (n=21) | 76.7% (n=69) |
| Veterinary Science 2 | 101 | 23.0 | 0.0% | 25.7% (n=26) | 74.3% (n=75) |
| Veterinary Science 3 | 52 | 24.0 (n51) | 26.9% (n=14) | 21.2% (n=11) | 78.8% (n=41) |

*Contributing % may not sum to 100% due to rounding or non-response; 1 University A, 2 University B, 3 University C; ^ Skewness = 3.322; Kurtosis = 14.145, minimum age 19 years, maximum age 58 years.

**Supplementary TABLE 2.** Demographic profile of respondents after data hygiene stages 1 and 2 (n = 594).

|  | Total | Median age | International % (n) | Male% (n) | Female % (n) |
| --- | --- | --- | --- | --- | --- |
| Engineering 1 | 60 | 22.0 | 36.7% (n=22) | 83.3% (n=50) | 16.7% (n=10) |
| Entrepreneurship 1 | 68 | 24.0 | 77.9% (n=53) | 52.9% (n=36) | 47.1% (n=32) |
| Nursing 1 | 176 | 21.0 (n=174) | 6.8% (n = 12) | 9.7% (n=17) | 90.3% (n=159) |
| Science 1 | 56 | 22.0 | 21.4% (n=12) | 58.9% (n=33) | 41.1% (n=23) |
| Veterinary Science (ALL) | 234 | 24.0 (n=233) | 8.1% (n=19) | 24.4% (n=57) | 75.6% (n=177) |
| **Total** | **594** | **23.0^ (n=591)** | **19.9% (n=118)** | **32.5% (n=193)** | **67.5% (n=401)** |
|  |  |  |  |  |  |
| Veterinary Science 1 | 86 | 24.0 | 8.1% (n=7) | 24.4% (n=21) | 75.6% (n=65) |
| Veterinary Science 2 | 98 | 23.0 | 0.0% | 25.5% (n=25) | 74.5% (n=73) |
| Veterinary Science 3 | 50 | 24.0 (n=49) | 24.0% (n=12) | 22.0% (n=11) | 78.0% (n=39) |

*Contributing % may not sum to 100% due to rounding or non-response; 1 University A, 2 University B, 3 University C, ^ Skewness = 3.329; Kurtosis = 14.545, minimum age 19 years, maximum age 58 years.

**Supplementary Table 3.** Estimated marginal means per gender and discipline for Other-Orientation and Self-Interest derived from the MANOVA (n = 586).

| Dependent Variable | Discipline | Mean | Std. Error | 95% Confidence Interval | |
| --- | --- | --- | --- | --- | --- |
|  |  |  |  | Lower Bound | Upper Bound |
| Other-Orientation | Male | .177 | .018 | .141 | .213 |
|  | Female | .217 | .017 | .184 | .249 |
|  | Engineer | .143 | .033 | .078 | .209 |
|  | Entrepreneurship | .179 | .030 | .119 | .238 |
|  | Nursing | .247 | .021 | .205 | .289 |
|  | Science | .232 | .033 | .167 | .297 |
|  | Veterinary Sci | .184 | .017 | .149 | .218 |
| Self-Interest | Male | .689 | .022 | .646 | .732 |
|  | Female | .633 | .020 | .594 | .672 |
|  | Engineer | .646 | .040 | .568 | .724 |
|  | Entrepreneurship | .693 | .036 | .622 | .764 |
|  | Nursing | .591 | .026 | .541 | .642 |
|  | Science | .683 | .039 | .605 | .760 |
|  | Veterinary Sci | .693 | .021 | .652 | .734 |
|  | | | | | |

| **Supplementary Table 4.** Pairwise comparisons of dependent variable means per gender and discipline from the simultaneous MANOVA of Other-Orientation and Self-Interest (n=586) | | | | | | | |  |
| --- | --- | --- | --- | --- | --- | --- | --- | --- |
| Dependent Variable | (I) Gender or Discipline | (J) Gender or Discipline | Mean Difference (I-J) | Std. Error | Sig.^d^ | 95% Confidence Interval for Difference^d^ | | |
|  |  |  |  |  |  | Lower Bound | Upper Bound | |
|  | Male | Female | -.039 | .025 | .119 | -.089 | .010 | |
| Other-Orientation | Female | Male | .039 | .025 | .119 | -.010 | .089 | |
|  | **Engineer** | Entrepreneurship | -.035 | .045 | .435 | -.124 | .053 | |
|  |  | **Nursing** | **-.104^*^** | **.042** | **.013** | **-.185** | **-.022** | |
|  |  | Science | -.089 | .047 | .057 | -.180 | .003 | |
|  |  | Veterinary Sci | -.040 | .039 | .301 | -.117 | .036 | |
|  | Entrepreneurship | Engineer | .035 | .045 | .435 | -.053 | .124 | |
|  |  | Nursing | -.068 | .037 | .067 | -.142 | .005 | |
|  |  | Science | -.054 | .045 | .233 | -.142 | .035 | |
|  |  | Veterinary Sci | -.005 | .035 | .883 | -.074 | .064 | |
|  | **Nursing** | **Engineer** | **.104^*^** | **.042** | **.013** | **.022** | **.185** | |
|  |  | Entrepreneurship | .068 | .037 | .067 | -.005 | .142 | |
|  |  | Science | .015 | .040 | .711 | -.064 | .093 | |
|  |  | **Veterinary Sci** | **.063^*^** | **.025** | **.012** | **.014** | **.113** | |
|  | **Science** | Engineer | .089 | .047 | .057 | -.003 | .180 | |
|  |  | Entrepreneurship | .054 | .045 | .233 | -.035 | .142 | |
|  |  | Nursing | -.015 | .040 | .711 | -.093 | .064 | |
|  |  | Veterinary Sci | .048 | .038 | .201 | -.026 | .123 | |
|  | **Veterinary Sci** | Engineer | .040 | .039 | .301 | -.036 | .117 | |
|  |  | Entrepreneurship | .005 | .035 | .883 | -.064 | .074 | |
|  |  | **Nursing** | **-.063^*^** | **.025** | **.012** | **-.113** | **-.014** | |
|  |  | Science | -.048 | .038 | .201 | -.123 | .026 | |
| Self-Interest | Male | Female | .056 | .030 | .062 | -.003 | .115 | |
|  | Female | Male | -.056 | .030 | .062 | -.115 | .003 | |
|  | Engineer | Entrepreneurship | -.047 | .054 | .382 | -.152 | .058 | |
|  |  | Nursing | .055 | .050 | .271 | -.043 | .152 | |
|  |  | Science | -.037 | .055 | .509 | -.145 | .072 | |
|  |  | Veterinary Sci | -.047 | .046 | .311 | -.138 | .044 | |
|  | **Entrepreneurship** | Engineer | .047 | .054 | .382 | -.058 | .152 | |
|  |  | **Nursing** | **.101^*^** | **.044** | **.023** | **.014** | **.189** | |
|  |  | Science | .010 | .054 | .848 | -.095 | .115 | |
|  |  | Veterinary Sci | .000 | .042 | .997 | -.082 | .082 | |
|  | Nursing | Engineer | -.055 | .050 | .271 | -.152 | .043 | |
|  |  | **Entrepreneurship** | **-.101^*^** | **.044** | **.023** | **-.189** | **-.014** | |
|  |  | Science | -.091 | .048 | .056 | -.185 | .002 | |
|  |  | **Veterinary Sci** | **-.102^*^** | **.030** | **<.001** | **-.160** | **-.043** | |
|  | Science | Engineer | .037 | .055 | .509 | -.072 | .145 | |
|  |  | Entrepreneurship | -.010 | .054 | .848 | -.115 | .095 | |
|  |  | Nursing | .091 | .048 | .056 | -.002 | .185 | |
|  |  | **Veterinary Sci** | -.010 | .045 | .817 | -.099 | .078 | |
|  | **Veterinary Sci** | Engineer | .047 | .046 | .311 | -.044 | .138 | |
|  |  | Entrepreneurship | .000 | .042 | .997 | -.082 | .082 | |
|  |  | **Nursing** | **.102^*^** | **.030** | **<.001** | **.043** | **.160** | |
|  |  | **Science** | .010 | .045 | .817 | -.078 | .099 | |
| Based on estimated marginal means | | | | | | | |  |
| *. The mean difference is significant at the .05 level. | | | | | | | |  |
| b. Adjustment for multiple comparisons: Least Significant Difference (equivalent to no adjustments). | | | | | | | |  |

|  | **Supplementary TABLE 5.** Stepwise addition of demographic and attitudinal covariates into the MULTIVARIATE linear regression model for standardized effect sizes [95% confidence intervals] for other-orientation and self-interest dependent variables estimated with the robust maximum likelihood (MLR) estimator (n = 583). | | | | | | |
| --- | --- | --- | --- | --- | --- | --- | --- |
|  | | Other-orientation | | | Self-Interest | | |
|  | | Model 1  ꞵ [95 CI] | Model 2  ꞵ [95 CI] | Model 3  ꞵ [95 CI] | Model 1  ꞵ [95 CI] | Model 2  ꞵ [95 CI] | Model 3  ꞵ [95 CI] |
| ***Demographic variables*** | |  |  |  |  |  |  |
| Gender (1 = women) | | .079 [-.006,.164] | .031 [-.062,.125] | .046 [-.136,.045] | **-.088 [-.172,-.005]** | **-.694 [-1.198,-.190]** | -.587 [-1.090,-.084] |
| AgeW32 | | .033 [-.050,.116] | .031 [-.052,.114] | .042 [-.035,.120] | -.038 [-.115,.039] | **-.146 [-.264,-.028]** | **-.145 [-.260,-.031]** |
| International (1=yes) | | -.061 [-.165,.042] | -.147 [-.295, .000] | **-.198 [-.338,-.058]** | -.065 [-.163,.033] | -.063 [-.160, .034] | -.039 [-.133,.055] |
| Entrepreneurship^ (1=yes) | | .072 [-.034,.179] | .073 [-.033,.178] | .097 [-.002,.196] | .098 [-.032,.228] | .108 [-.021,.236] | .088 [-.040,.215] |
| Nursing^ (1=yes) | | **.180 [.048,.312]** | **.186 [.055,.317]** | **.129 [.007,.252]** | **-.069 [-.226,-.088]** | -.045 [-.202,.113] | -.001 [-.150,.147] |
| Science^ (1=yes) | | **.109 [.019,.199]** | .101 [.013,.189] | .119 [.037,.201] | .049 [-.057,.155] | .052 [-.054,.158] | .035 [-.070,.139] |
| Veterinary^ (1=yes) | | **.211 [.033,.390]** | .223 [.046,.400] | **.229 [.067,.390]** | -.062 [-.263,.139] | -.053 [-.254,.149] | -.058 [-.256,.141] |
| University 2^#^ (1=yes) | | **-.215 [-.334,-.095]** | **-.219 [-.338,-.100]** | **-.231 [-.343,-.119]** | **.169 [.056,.281]** | **.169 [.056,.282]** | **.177 [.068,.285]** |
| University 3^#^ (1=yes) | | -.069 [-.163,.026] | -.078 [-.171, .014] | -.078 [-.162, .006] | **.176 [.088,.265]** | **.173 [.085,.262]** | **.172 [.086,.257]** |
| ***Demographic variable Interactions*** | | |  |  |  |  |  |
| Gender*AgeW32 | |  |  |  |  | **.603 [.097,1.109]** | .546 [.043,1.048] |
| Gender*International | |  | .120 [.008,.233] | .151 [.040,.262] |  |  |  |
| ***Attitudinal effects*** | |  |  |  |  |  |  |
| Prosociality | |  |  | **.319 [ .248,.390]** |  |  | **-.250 [-.331,-.169]** |
| Income Importance | |  |  | **-.189 [-.264,-.114]** |  |  | **.133 [.047,.219]** |
| ***Variance explained*** | |  |  |  |  |  |  |
| R^2^ | | **.061 (*p*=.002)** | **.067 (*p* =.002)** | **.194 (*p* =.000)** | **.063 (*p*=.000)** | **.070 (*p* =.000)** | **.147 (*p* =.000)** |
| ∆R^2^ | | . | .006 | .127 | . | .040 | .077 |
| ***Changes between models*** | | | | | | | |
| OO SI correlation in Model 1 | |  |  |  | **-.483 [-.570,-.397]** |  |  |
| OO SI correlation in Model 2 | |  |  |  |  | **-.489 [-.572,-.405]** |  |
| OO SI correlation in Model 3 | |  |  |  |  |  | **-.432 [-.518,-.345]** |
| ∆χ^2^ Model 2 from Model 1 | |  |  |  |  | **Chi2(4) = 11.507, p<.025** |  |
| ∆χ^2^ Model 3 from Model 2 | |  |  |  |  |  | **Chi2(4) = 89.436, p<.001** |
| OO Other-Orientation; SI Self-Interest, R^2^ variance explained in the dependent variable; ∆R^2^ the increase in the variance explained to the previous model. AgeWindsorized; ^ referent is Engineering; ^#^ only veterinary respondents; ∆χ^2^ TRd test statistic (https://www.statmodel.com/chidiff.shtml) | | | | | | | |

Both gender and age did not affect other-orientation but both gender (being female) and age (being older) negatively affected self-interest in Model 3. Further, gender and age interacted together to affect self-interest.

International student status did not affect self-interest. However, international student status directly affected Other-Orientation, and in a two-way interaction with gender, positively affected Other-orientation.

Being an entrepreneurship respondent (compared to an engineering respondent) did not directly affect other-orientation or self-interest in any of the models. While being a nursing, science, or veterinary student (compared to engineering respondents) positively affected other-orientation in all models, but not self-interest.

Being a respondent from University 2 (i.e., a veterinary respondent from University 2) negatively affected other-orientation and positively affected self-interest in all models. In contrast, being a respondent from University 3 (i.e., a veterinary respondent from University 3) had no effect on other-orientation, but positively contributed to self-interest of a respondent.

Prosociality of a respondent positively affected other-orientation, but negatively affected self-interest. Income Importance to a respondent negatively affected other-orientation but positively affected self-interest.

**Supplementary TABLE 6**. Sample and cluster mean scores (standard errors) for the four-profile K-means cluster results (N = 586).

| Variables | Total Sample  (n=586)  (100%) | Cluster | | | |
| --- | --- | --- | --- | --- | --- |
|  |  | 1  (*n*=51)  8.7% | 2  (*n*=199)  34.0% | 3  (*n*=285)  48.6% | 4  (*n*=51)  8.7% |
| Other-Orientation | .21 (.01) | .55 (.03) | .40 (.01) | .10 (.01) | -.26 (.02) |
| Self-Interest | .65 (.01) | -.05 (.04) | .54 (.01) | .87 (.01) | .56 (.03 |

**Supplementary TABLE 7.** Level of support for hypotheses.

| **No.** | **Hypotheses** | **Supported or unsupported** |
| --- | --- | --- |
| 1a | The level of other-orientation for veterinary respondents will be more similar to respondents in another patient-focussed discipline (nursing) and less similar to those in non-health-care disciplines. | Not-Supported |
| 1b | The level of self-interest for veterinary respondents will be more similar to respondents in another patient-focussed discipline (nursing) and less similar to those in non-health-care disciplines. | Not supported |
| 2a | The level of other-orientation for women will be greater than for men in our study population. | Not Supported |
| 2b | The level of self-interest for women will be less than for men in our study population. | Not Supported |
| 3a | The level of other-orientation for women and men veterinary respondents will be similar. | Supported |
| 3b | The level of self-interest for men veterinary respondents will be greater than for women veterinary respondents. | Not-Supported |
| 4 | Our study population will have distinct interpretable profiles of different combinations of other-orientation and self-interest, including a profile of individuals who are both other-oriented and self-interested. | Supported |
| **5** | **There will be differences in the proportional representation of identified cluster-based profiles of veterinary respondents to respondents of other disciplines.** | **Supported.** |
| **6** | **There will be differences in the proportional representation of identified cluster-based profiles of veterinary respondents from different institutions.** | **Supported.** |
